# Pyruvate Facilitates FACT‐Mediated *γ*H2AX Loading to Chromatin and Promotes the Radiation Resistance of Glioblastoma

**DOI:** 10.1002/advs.202104055

**Published:** 2022-01-20

**Authors:** Siyang Wu, Ruixiu Cao, Bangbao Tao, Ping Wu, Chao Peng, Hong Gao, Ji Liang, Weiwei Yang

**Affiliations:** ^1^ State Key Laboratory of Cell Biology Shanghai Key Laboratory of Molecular Andrology Shanghai Institute of Biochemistry and Cell Biology Center for Excellence in Molecular Cell Science Chinese Academy of Sciences University of Chinese Academy of Sciences Shanghai 200031 China; ^2^ School of Life Science Hangzhou Institute for Advanced Study University of Chinese Academy of Sciences Hangzhou 310024 China; ^3^ Department of Neurosurgery XinHua Hospital School of Medicine Shanghai Jiaotong University Shanghai 200092 China; ^4^ National Facility for Protein Science in Shanghai Zhangjiang Lab Shanghai Advanced Research Institute Chinese Academy of Science Shanghai 201210 China; ^5^ Shanghai Science Research Center Chinese Academy of Sciences Shanghai 201204 China

**Keywords:** FACT complex, glioblastoma, PKM2, pyruvate, radioresistance

## Abstract

DNA repair confers the resistance of tumor cells to DNA‐damaging anticancer therapies, while how reprogrammed metabolism in tumor cells contributes to such process remains poorly understood. Pyruvate kinase M2 isoform (PKM2) catalyzes the conversion of phosphoenolpyruvate to pyruvate and regulates the last rate‐limiting step of glycolysis. Here it is shown that the glycolytic metabolite pyruvate enhances DNA damage repair by facilitating chromatin loading of *γ*H2AX, thereby promoting the radiation resistance of glioma cells. Mechanistically, PKM2 is phosphorylated at serine (S) 222 upon DNA damage and interacts with FACT complex, a histone chaperone comprising SPT16 and SSRP1 subunit. The pyruvate produced by PKM2 directly binds to SSRP1, which increases the association of FACT complex with *γ*H2AX and subsequently facilitates FACT‐mediated chromatin loading of *γ*H2AX, ultimately promoting DNA repair and tumor cell survival. Intriguingly, the supplementation of exogenous pyruvate can also sufficiently enhance FACT‐mediated chromatin loading of *γ*H2AX and promotes tumor cell survival upon DNA damage. The levels of PKM2 S222 phosphorylation correlate with the malignancy and prognosis of human glioblastoma. The finding demonstrates a novel mechanism by which PKM2‐produced pyruvate promotes DNA repair by regulating *γ*H2AX loading to chromatin and establishes a critical role of this mechanism in glioblastoma radiation resistance.

## Graphical abstract

DNA repair confers the resistance of tumor cells to DNA‐damaging anticancer therapies, while how reprogrammed metabolism in tumor cells contributes to the process of DNA repair remains poorly understood. Wu et al. demonstrate here that the glycolytic metabolite pyruvate facilitates the FACT complex‐mediated *γ*H2AX loading to chromatin and enhances repair of damaged DNA, thereby promoting radiation resistance of glioblastoma.

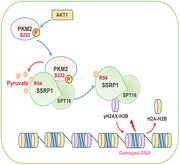

## 1 Introduction

Therapeutic resistance continues to be an indomitable foe in our ambition for curative cancer treatment. Multiple sets of mechanisms have been uncovered to be involved in such resistance of cancer treatment, such as deregulated drug transport, decreased cell death, enhanced DNA repair, and mutations in drug targets.^[^ ^1^, ^2^ ^]^ Since many classical cancer therapies cause DNA damage, the importance of dysregulated DNA repair system in the resistance of cancer treatment has been increasingly recognized. The use of inhibitors targeting DNA repair machinery or DNA damage signaling pathways could provide important opportunities to improve the efficacy of classical chemo‐ and radiotherapies by overcoming the therapeutic resistance.^[^ ^3^, ^4^ ^]^

Deregulated glycolysis, also known as Warburg effect, commonly exists in most cancers, exhibiting increased glucose uptake and lactate production.^[^ ^5^ ^]^ By providing the precursors for the biosynthesis of nucleotides, amino acids, and lipids, high rate of glycolysis allows the cells to maintain biosynthetic fluxes during rapid proliferation.^[^ ^6^ ^]^ In addition, the glycolytic pathway can also be diverted to the pentose phosphate pathway, which generates NADPH for the antioxidant activity of glutathione, thereby sustaining redox homeostasis and supporting tumor cell survival.^[^ ^7^ ^]^ Intriguingly, emerging evidence suggests that enhanced glycolysis is associated with the resistance of tumor cells to radiation partly by enhancing DNA damage repair.^[^ ^8^, ^9^, ^10^ ^]^ However, it remains unclear how glycolysis, especially the glycolytic metabolites, regulates DNA repair machinery.

Pyruvate kinase (PK) transfers a phosphate group from phosphoenolpyruvate (PEP) to ADP to generate ATP and pyruvate, thereby regulating the final rate‐limiting step of glycolysis. PKM2 is a splicing variant of pyruvate kinase overexpressed in many types of cancers and plays an important role in tumor cell proliferation because of its function in Warburg effect.^[^ ^11^, ^12^ ^]^ In addition to its metabolic function, nonmetabolic functions of PKM2 have also been extensively addressed in many recent studies, in which PKM2 can function as a coactivator of transcription factor or a protein kinase that phosphorylates transcription factors, histones and cell cycle regulator to modulate gene expression, chromatin remodeling, metabolic rewiring, and cell cycle progression.^[^ ^11^, ^13^, ^14^, ^15^, ^16^ ^]^

In this study, we investigated the role and regulatory mechanism of PKM2 in DNA repair system in tumor cells. We reveal a new role of pyruvate to enhance DNA repair in tumor cells by regulating *γ*H2AX loading to chromatin and demonstrate the regulatory mechanism underlying such role of pyruvate.

## 2 Results

### 2.1 PKM2 Is Required for DNA Repair and Tumor Cell Survival upon DNA Damage

**Figure 1:**
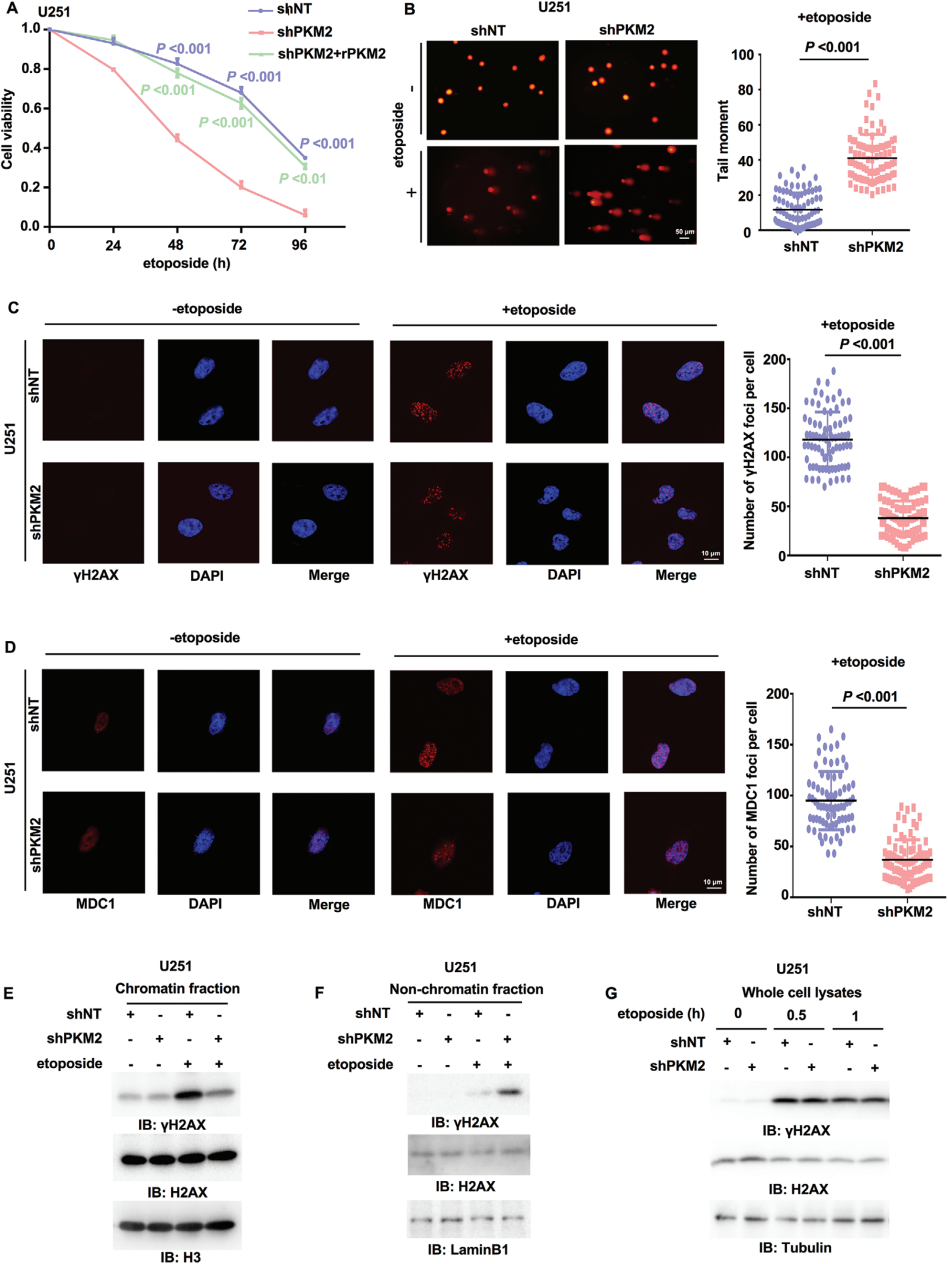
PKM2 is required for *γ*H2AX levels in chromatin, DNA repair, and tumor cell survival. Immunoblotting (IB) experiments were performed with the indicated antibodies. Data are representative of at least three independent experiments. A) U251 cells stably expressing a nontargeting shRNA or PKM2 shRNA were rescued with or without rPKM2. Cells were treated with etoposide (200 × 10^–6^ m) for indicated time. Cell viability was determined using Trypan blue staining. Data represent the mean ± SD of the viability of the cells from three independent experiments (two‐tailed Student's t‐test). *P* values for comparisons between shNT and shPKM2 are shown in blue; comparisons between shPKM2 and shPKM2+rPKM2 are shown in green. B) U251 cells stably expressing shNT or shPKM2 were treated with or without etoposide (500 × 10^–6^ m) for 3 h. The representative images of the comet assay in these cells were shown (left panel). Scatter dot plot (right panel) of the tail moments in the comet assay from shNT‐expressing cells (*n* = 86) or shPKM2‐expressing cells (*n* = 86) treated with etoposide. Data represent mean ± SD of the tail moments (Mann Whitney test). Data are representative of three independent experiments. C,D) U251 cells stably expressing shNT or shPKM2 were treated with or without etoposide (40 × 10^–6^ m, 0.5 h). C) IF staining was performed using anti‐*γ*H2AX antibody. Representative images were shown (left panel). Scatter dot plot (right panel) represents the number of *γ*H2AX foci per cell. Data represent the mean ± SD of the number of *γ*H2AX foci from shNT‐expressing cells (*n* = 80) and shPKM2‐expressing cells (*n* = 81) treated with etoposide (Mann Whitney test). Data are representative of three independent experiments. D) IF staining was performed using anti‐MDC1 antibody. Representative images were shown (left panel). Scatter dot plot (right panel) represents the number of MDC1 foci per cell. Data represent mean ± SD of the MDC1 foci number of shNT‐expressing cells (*n* = 75) or shPKM2‐expressing cells (*n* = 81) treated with etoposide (Mann Whitney test). Data are representative of three independent experiments. E,F) U251 cells with or without PKM2 depletion were treated with or without etoposide (200 × 10^–6^ m, 1 h). E) The chromatin fraction and F) nonchromatin‐bound fraction were prepared. G) U251 cells stably expressing shNT or shPKM2 were treated with or without etoposide (200 × 10^–6^ m) for indicated time. Whole‐cell lysates were prepared after sonication.

To investigate the role of PKM2 in DNA repair in tumor cells, we depleted endogenous PKM2 in U251 and U87 human glioblastoma multiforme (GBM) cells by infecting a lentivirus expressing a specific shRNA against PKM2 and rescued these cells with or without shRNA‐resistant (r) PKM2 (Figure S1A, Supporting Information), followed by the treatment of etoposide, the specific inhibitor of Topoisomerase II which can generate DNA double‐strand breaks. PKM2 depletion dramatically decreased the viability of tumor cells after etoposide treatment. Notably, rescued expression of rPKM2 almost completely restored the viability to the levels of shNT‐expressing cells (**Figure** **1**A and Figure S1B, Supporting Information), excluding the off‐targeting possibility of shPKM2. Moreover, as determined by comet assay, PKM2 depletion greatly aggravated etoposide‐induced DNA damage in tumor cells (Figure 1B and Figure S1C, Supporting Information). Together, these results implicate that PKM2 promotes cell survival likely by promoting DNA repair.

### 2.2 PKM2 Depletion Decreases *γ*H2AX Levels in Chromatin and Inhibits DNA Repair Foci Formation

DNA damage could elicit chromatin remodeling, mainly including histone modifications, histone degradation, and incorporation of histone variants,^[^ ^17^, ^18^ ^]^ that are executed by histone‐modifying enzymes, ATP‐dependent remodeling enzymes and histone chaperones.^[^ ^19^ ^]^ One of the earliest cellular responses introduced by DNA damage is the phosphorylation of H2A variant H2AX serine (S) 139 (*γ*H2AX) at double‐strand break (DSB) within a few minutes and evoke the following aggregation and spreading of repair factors. Mediator of DNA damage checkpoint protein 1 (MDC1) serves as a scaffold for the recruitment of DNA repair and signal transduction proteins to discrete foci of DNA damage marked by *γ*H2AX.^[^ ^20^ ^]^

To confirm the regulation of DNA repair by PKM2, we next determine whether PKM2 regulates DNA repair foci formation. We performed immunofluorescence (IF) staining with anti‐*γ*H2AX and anti‐MDC1 antibodies, which showed that PKM2 depletion greatly decreased the number of etoposide‐induced *γ*H2AX foci and MDC1 foci in U251 and U87 cells (Figure 1C,D and Figure S1D, Supporting Information). In addition, we carried out subcellular fractionation in U251 and U87 cells treated with etoposide. Immunoblotting analysis of *γ*H2AX showed that PKM2 depletion decreased the levels of *γ*H2AX in chromatin‐bound fraction but meanwhile increased the levels of *γ*H2AX in nonchromatin‐bound fraction after etoposide treatment (Figure 1E,F and Figure S1E–G, Supporting Information). Notably, the levels of total *γ*H2AX were not influenced by PKM2 depletion (Figure 1G). Taken together, these results demonstrate that PKM2 regulates *γ*H2AX levels in chromatin and promotes DNA repair foci formation.

### 2.3 PKM2 Interacts with FACT and Enhances the Interaction between FACT and Nonchromatin Bound *γ*H2AX

**Figure 2:**
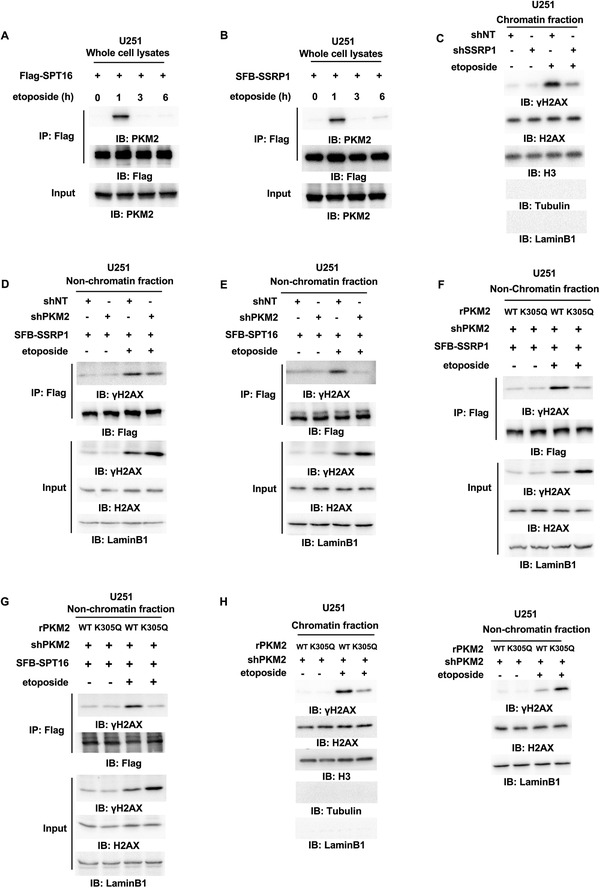
PKM2 interacts with FACT and enhances the interaction between FACT and nonchromatin bound *γ*H2AX. Immunoprecipitation (IP) and immunoblot (IB) analyses were performed with indicated antibodies. Data are representative of at least three independent experiments. A,B) U251 cells were transfected with A) Flag‐SPT16 or B) SFB‐SSRP1 and treated with etoposide (200 × 10^–6^ m) for indicated time. Whole‐cell lysates were prepared without sonication. C) U251 cells with or without SSRP1 depletion were treated with or without etoposide (200 × 10^–6^ m, 1 h). The chromatin fraction was prepared. D,E) U251 cells stably expressing shNT or shPKM2 were infected with the lentivirus expressing D) SFB‐SSRP1 or E) SFB‐SPT16 and treated with or without etoposide (200 × 10^–6^ m, 1 h). The nonchromatin‐bound fraction was prepared. F,G) PKM2‐depleted U251 cells were rescued with rPKM2 WT or K305Q and infected with the lentivirus expressing F) SFB‐SSRP1 or G) SFB‐SPT16. The cells were treated with or without etoposide (200 × 10^–6^ m, 1 h) and the nonchromatin‐bound fraction was prepared. H) PKM2‐depleted U251 cells were rescued with rPKM2 WT or K305Q followed by etoposide treatment (200 × 10^–6^ m, 1 h). Chromatin fraction (left panel) and nonchromatin‐bound fraction (right panel) were prepared.

To investigate how PKM2 regulates *γ*H2AX levels in chromatin, we performed a mass spectrometry analysis of PKM2‐associated proteins in U251 cells after etoposide treatment. SPT16, the subunit of FACT complex, was identified to have strong interaction with PKM2 in U251 cells after etoposide treatment (Figure S2A, Supporting Information). The FACT complex, a general chromatin factor that acts to reorganize nucleosomes, is involved in multiple processes, such as mRNA elongation, DNA replication, and DNA repair.^[^ ^21^, ^22^ ^]^ During transcription elongation, the FACT complex facilitates the passage of RNA polymerase II and transcription by promoting the dissociation of one histone H2A‐H2B dimer from the nucleosome, then subsequently promotes the reestablishment of the nucleosome following the passage of RNA polymerase II.^[^ ^23^ ^]^ We validated the interaction between these two proteins by coimmunoprecipitation (co‐IP) experiment in U251 cells transfected with Flag‐tagged SPT16 (Flag‐SPT16) after etoposide (**Figure** **2**A). Moreover, SSRP1, the other subunit of FACT complex, was also observed to interact with PKM2 after etoposide treatment (Figure 2B), suggesting that PKM2 interacts with the FACT complex upon DNA damage.

In addition, we performed subcellular fractionation and immunofluorescence in U251 or U87 cells treated with or without etoposide, which showed that etoposide treatment increases the nuclear localization of PKM2 in these cells (Figure S2B,C, Supporting Information). Moreover, the interaction between PKM2 and SSRP1 was observed in the nucleus, but not in the cytosol after etoposide treatment (Figure S2D, Supporting Information).

To test whether the FACT complex contributes to DNA damage‐induced *γ*H2AX in chromatin, we depleted SSRP1 in U251 cells and found that SSRP1 depletion significantly attenuated the levels of *γ*H2AX in chromatin (Figure 2C and Figure S2E, Supporting Information), suggesting that the FACT complex is essential for *γ*H2AX levels in chromatin in response to DNA damage.

We next investigated how PKM2 regulates *γ*H2AX levels in chromatin through the FACT complex. U251 cells stably expressing S peptide‐flag‐streptavidin binding peptide (SFB)‐SSRP1 or SFB‐SPT16 were infected with the lentivirus expressing shNT or shPKM2 and treated with or without etoposide. Co‐IP experiments with anti‐Flag antibody in these cells showed that PKM2 depletion dramatically attenuated etoposide‐induced interaction between FACT and *γ*H2AX in nonchromatin bound fraction (Figure 2D,E).

Intriguingly, we generated enzymatic activity‐dead mutant of PKM2, PKM2 K305Q, in which lysine (K) 305 was mutated to glutamine (Q).^[^ ^24^ ^]^ PKM2 K305Q had much lower activity than PKM2 WT (Figure S2F, Supporting Information). PKM2‐depleted U251 cells were then rescued with rPKM2 WT or K305Q (Figure S2G, Supporting Information). As shown in Figure 2F,G, the expression of rescued rPKM2 K305Q dramatically decreased the interaction between the FACT subunit (SSRP1 or SPT16) and *γ*H2AX in nonchromatin bound fraction after etoposide treatment. Moreover, the levels of *γ*H2AX in chromatin were also decreased by the expression of rescued rPKM2 K305Q and meanwhile the levels of *γ*H2AX in nonchromatin bound fraction were increased (Figure 2H). Notably, PKM2 in nuclear fraction from U87 cells treated with etoposide was immunoprecipitated and examined for its glycolytic activity in the absence or presence of Shikonin (the inhibitor of PKM2), which showed that nuclear PKM2 still kept its glycolytic activity, which could be inhibited by Shikonin treatment (Figure S2H, Supporting Information). To determine the oligomerization state of nuclear PKM2, PKM2 was immunoprecipitated in nuclear fraction of U87 cells expressing Flag‐PKM2 after etoposide treatment, followed by gel filtration analysis, which showed that nuclear PKM2 existed as both tetramer and dimer (Figure S2I, Supporting Information).

Collectively, these results demonstrate that PKM2 is translocated into nucleus upon DNA damage, where it interacts with the FACT complex. PKM2, especially its glycolytic activity, is required for the interaction between FACT and *γ*H2AX in nonchromatin bound fraction and ultimate *γ*H2AX levels in chromatin after etoposide treatment.

### 2.4 Pyruvate Increases the Interaction between FACT and *γ*H2AX, *γ*H2AX Levels in Chromatin and Tumor Cell Survival upon DNA Damage

**Figure 3:**
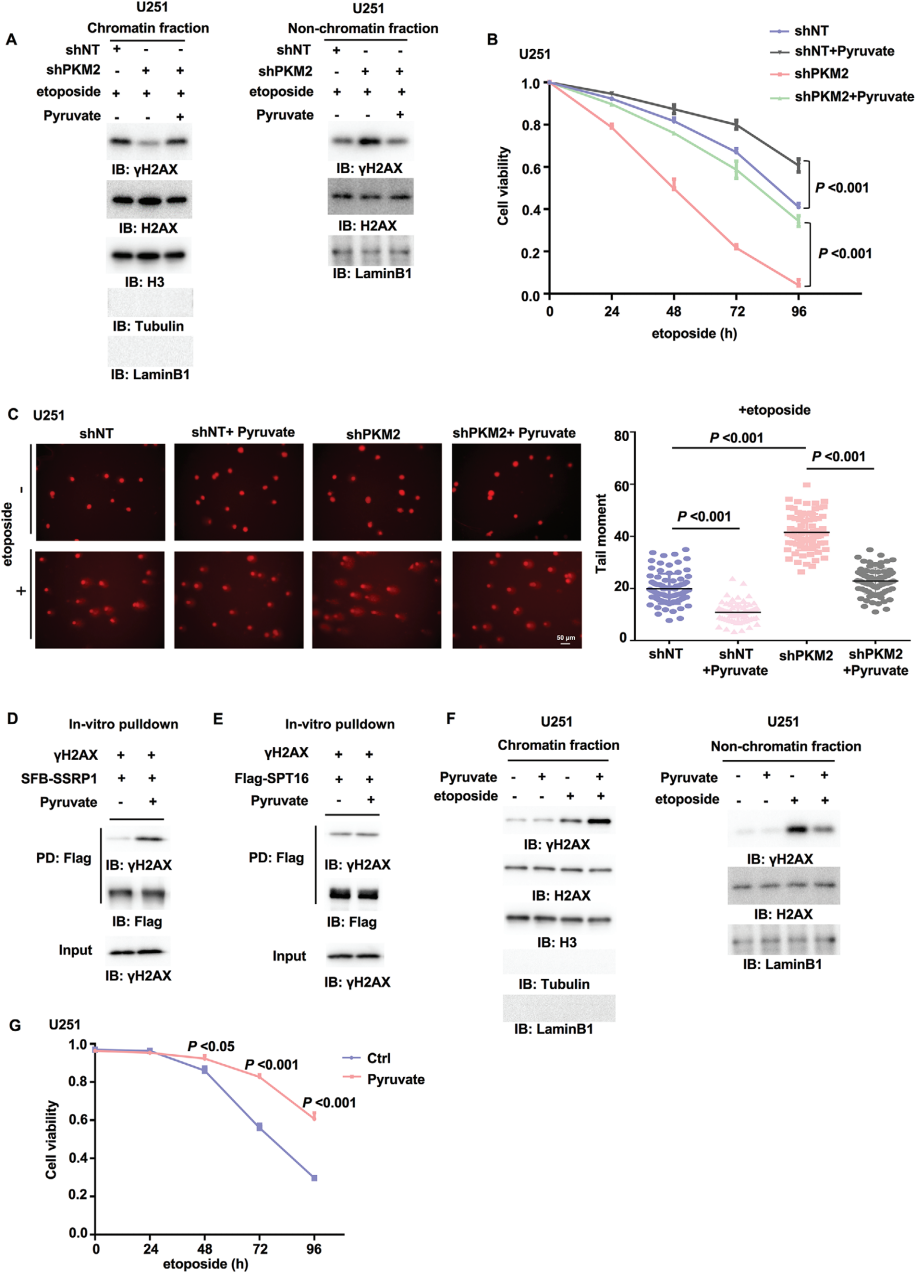
Pyruvate increases the interaction between FACT and *γ*H2AX, *γ*H2AX levels in chromatin and tumor cell survival upon DNA damage. IP and IB analyses were performed with indicated antibodies. Data are representative of at least three independent experiments. A) U251 cells stably expressing shNT or shPKM2 were supplemented with or without pyruvate (10 × 10^–3^ m, 3 h) and then treated with etoposide (200 × 10^–6^ m, 1 h). Chromatin fraction (left panel) and nonchromatin‐bound fraction (right panel) were prepared. B) U251 cells stably expressing shNT or shPKM2 were supplemented with or without pyruvate (10 × 10^–3^ m, 3 h) and then treated with etoposide (200 × 10^–6^ m) for indicated time. Cell viability was determined. Data represent the mean ± SD of the viability of the cells from three independent experiments (two‐tailed Student's t‐test). C) U251 cells stably expressing shNT or shPKM2 were supplemented with or without pyruvate (10 × 10^–3^ m, 3 h) and then treated with or without etoposide (500 × 10^–6^ m) for 3 h. The representative images of the comet assay in these cells were shown (left panel). Scatter dot plot (right panel) represents the tail moments in the comet assay from cells treated with etoposide, expressing shNT with (*n* = 75) or without (*n* = 83) pyruvate, or cells expressing shPKM2 with (*n* = 78) or without (*n* = 78) pyruvate. Data represent mean ± SD of the tail moments (Mann Whitney test). Data are representative of three independent experiments. D,E) SSRP1 or SPT16 was immunoprecipitated and purified from U251 cells stably expressing D) SFB‐SSRP1 or E) Flag‐SPT16. *γ*H2AX was immunoprecipitated and purified from etoposide‐treated U251 cells. The in vitro pulldown experiment was performed by incubating the purified SSRP1 or SPT16 with purified *γ*H2AX in the absence or presence of pyruvate (1 × 10^–3^ m). PD: pulldown. F) U251 cells were supplemented with pyruvate (10 × 10^–3^ m, 3 h) and then treated with etoposide (200 × 10^–6^ m, 1 h). Chromatin fraction (left panel) and nonchromatin‐bound fraction (right panel) were prepared. G) U251 cells were supplemented with or without pyruvate (10 × 10^–3^ m, 3 h) and then treated with etoposide (200 × 10^–6^ m) for indicated time. Cell viability was determined using Trypan blue staining. Data represent the mean ± SD of the viability of the cells from three independent experiments (two‐tailed Student's t‐test).

Since PKM2 activity is required for DNA damage‐induced *γ*H2AX in chromatin, we determined whether PKM2 regulates *γ*H2AX through its product pyruvate. The supplementation of exogenous pyruvate greatly restored the levels of *γ*H2AX in chromatin and cell survival in PKM2‐depleted U251 cells after etoposide treatment (**Figure** **3**A,B), suggesting that pyruvate is required for PKM2‐regulated *γ*H2AX levels in chromatin and cell survival upon DNA damage. Additionally, as shown in Figure 3C and Figure S3A (Supporting Information), the supplementation of exogenous pyruvate also greatly enhanced DNA repair (indicated by MDC1 foci) and attenuated DNA damage in PKM2‐depleted U251 cells after etoposide treatment. Of note, the supplementation of exogenous pyruvate could further enhance DNA repair and attenuate etoposide‐induced DNA damage in U251 cells expressing shNT.

Emerging evidence demonstrates that metabolite not only functions in metabolic pathway as a metabolic intermediate, but also acts as a signaling molecule to regulate the epigenetics and signal transduction.^[^ ^25^, ^26^ ^]^ We next explored how pyruvate regulates *γ*H2AX levels in chromatin. We purified SSRP1, SPT16, or *γ*H2AX from etoposide‐treated U251 cells and mixed these proteins with or without pyruvate or ATP. The pulldown assay showed that pyruvate, but not ATP, dramatically increased the interaction between SSRP1 and *γ*H2AX but only slightly enhanced the interaction between SPT16 and *γ*H2AX (Figure 3D,E and Figure S3B, Supporting Information), implicating that pyruvate directly regulate the interaction between SSRP1 and *γ*H2AX. Then we wondered whether the glycolytic activity and subsequent pyruvate production are altered by DNA damage treatment, thereby regulating the interaction between FACT and *γ*H2AX. As shown in Figure S3C (Supporting Information), etoposide treatment did not change the glycolytic activity and the levels of total pyruvate in U251 cells. The examination of pyruvate levels in cytosol and nucleus showed that the levels of pyruvate in nucleus were increased after etoposide treatment (Figure S3D, Supporting Information). However, no obvious decrease was detected in the levels of cytosolic pyruvate after etoposide, which might be due to the much higher pyruvate levels in cytosol than those in nucleus. Therefore, it may be difficult to detect such a slight decrease of pyruvate levels in cytosol. Along with the fact that PKM2 interacts with FACT, we hypothesized that PKM2 might provide a local source of pyruvate for FACT by interacting with FACT.

Intriguingly, the supplementation of extra exogenous pyruvate could further increase *γ*H2AX levels in chromatin and meanwhile decreased *γ*H2AX levels in nonchromatin bound fraction in U251 and U87 cells after etoposide treatment (Figure 3F and Figure S3E, Supporting Information). Functionally, the viability of tumor cells treated with etoposide was increased by exogenous pyruvate (Figure 3G and Figure S3F, Supporting Information). In addition, we cultured U251 cells with low glucose or high glucose media supplemented with or without pyruvate. As shown in Figure S3G (Supporting Information), etoposide induced more DNA damage in the cells under low glucose than in the cells under high glucose, as determined by comet assay. The supplementation of pyruvate attenuated etoposide‐induced DNA damage in the cells under low glucose. We determined the levels of pyruvate in these cells and found that the cells under high glucose contained higher pyruvate levels than the cells under low glucose. And pyruvate supplementation greatly restored the pyruvate levels in the cells under low glucose (Figure S3H, Supporting Information). Collectively, these results indicate that pyruvate sufficiently enhances DNA repair and promotes tumor cell survival upon DNA damage by regulating *γ*H2AX levels in chromatin.

Taken together, these results suggest that either the interaction of PKM2 with FACT or supplementation of exogenous pyruvate is able to provide sufficient pyruvate to enhance the interaction between FACT and *γ*H2AX, thereby promoting FACT‐mediated chromatin loading of *γ*H2AX and DNA repair.

### 2.5 Pyruvate Directly Binds to SSRP1

**Figure 4:**
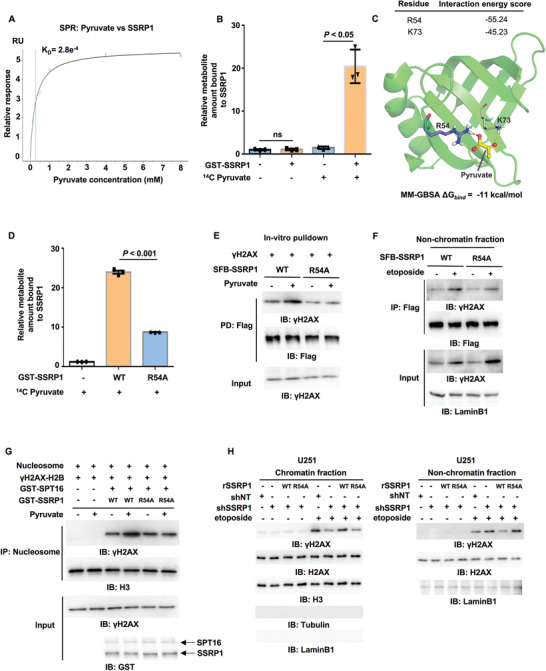
The binding of pyruvate to SSRP1 is required for FACT‐mediated *γ*H2AX loading to chromatin. IP and IB analyses were performed with indicated antibodies. Data are representative of at least three independent experiments. A) SPR (surface plasmon resonance) assay was performed with bacteria‐purified recombinant SSRP1 protein (100 µg mL^–1^) and increasing doses of pyruvate. *K*_D_: dissociation constant (pyruvate versus SSRP1). B) Metabolite–protein binding assay. Bacteria‐purified recombinant GST‐SSRP1 was incubated with ^14^C‐labeled pyruvate. SSRP1‐bound pyruvate was quantified by scintillation counting. Data represent the mean ± SD of the relative metabolite amount bound to SSRP1 from three independent experiments (two‐tailed Student's t‐test). C) A representative image of the structure of human SSRP1 bound to pyruvate was shown. Atoms of pyruvate are presented as balls and sticks with carbon atoms in yellow and oxygen atoms in red. The whole protein is shown as a cartoon in green, while R54 is shown as sticks with purple carbon atoms and K73 is shown as lines with gray carbon atoms. Red dashed lines represent salt‐bridge and yellow ones for hydrogen bonds. The binding energy (Δ*G*_bind_) of pyruvate with SSRP1 calculated using MM‐GBSA is ‐11 kcal mol^–1^. D) Metabolite–protein binding assay. Bacteria‐purified recombinant GST‐SSRP1 WT or R54A was incubated with ^14^C‐labeled pyruvate. The pyruvate bound to SSRP1 WT or R54A was quantified by scintillation counting. Data represent the mean ± SD of the relative metabolite amount bound to SSRP1 from three independent experiments (two‐tailed Student's t‐test). E) SSRP1 WT or R54A was immunoprecipitated and purified from U251 cells stably expressing SFB‐SSRP1 WT or R54A. *γ*H2AX was immunoprecipitated and purified from etoposide‐treated U251 cells. The in vitro pulldown experiment was performed by incubating the purified SSRP1 WT or R54A with purified *γ*H2AX in the absence or presence of pyruvate (1 × 10^–3^ m). F) HEK293T cells were transfected with SFB‐SSRP1 WT or R54A and then treated with or without etoposide (200 × 10^–6^ m, 1 h). Nonchromatin‐bound fraction was prepared. G) In vitro histone exchange assay. H2AX‐H2B dimers were phosphorylated by purified recombinant monomeric ATM to obtain *γ*H2AX‐H2B dimers. Commercially purchased nucleosomes (120 ng) were incubated with *γ*H2AX‐H2B dimers, GST‐SPT16 and GST‐SSRP1 WT or R54A in the absence or presence of pyruvate (1 × 10^–3^ m) for 1 h. The recruitment of *γ*H2AX to the chromatin was determined by IB analysis of immobilized nucleosomes with anti‐*γ*H2AX antibody. Histone H3 was used as loading control. H) U251 cells were infected with the lentivirus expressing shNT or shSSRP1 and the SSRP1‐depleted U251 cells were reconstituted with or without the expression of rSSRP1 WT or R54A. These cells were then treated with or without etoposide (200 × 10^–6^ m, 1 h). Chromatin fraction (left panel) and non‐chromatin‐bound fraction (right panel) were prepared.

To explore how pyruvate increases the interaction between FACT and *γ*H2AX, we first test whether pyruvate directly binds to the FACT complex. Because pyruvate only slightly increases the interaction between SPT16 and *γ*H2AX (Figure 3E), we wondered whether pyruvate directly interacted with SSRP1. To address this question, we performed surface plasmon resonance (SPR) with recombinant human SSRP1 and pyruvate, which confirmed the direct interaction between pyruvate and SSRP1 (**Figure** **4**A). This result was also supported by radiometric metabolite–protein interaction assay with ^14^C‐labeled pyruvate (Figure 4B).

To map the interface in SSRP1 that is associated with pyruvate, we performed limited proteolysis‐small molecule mapping (LiP‐SMap) by mixing pyruvate and recombinant SSRP1. A peptide ranging from residue 46 to residue 55 in SSRP1 was identified to interact with pyruvate (Figure S4A, Supporting Information). To further pinpoint the exact residues in SSRP1 that are required for their interaction, we carried out molecular docking, in which the protein receptor (SSRP1) was prepared based on the crystal structure of the N‐terminal domain (residue 1–100) of human SSRP1 (PDB ID: 5UMR) by Protein Preparation Wizard and the ligands (pyruvate) were built and prepared by LigPrep. Then pyruvate was docked to SSRP1 at the SP precision by Glide with default parameters. The binding free energy (Δ*G*_bind_) of pyruvate with SSRP1, calculated by MM‐GBSA, was ‐11 kcal mol^–1^. R54 and K73 in SSRP1 were the dominant residues for pyruvate binding to SSRP1. The interaction energy score of R54 and K73 with pyruvate are ‐55.24 and ‐45.23, respectively (Figure 4C).

Basing on these two results, we speculated that R54 is required for SSRP1 interaction. To test this hypothesis, we generated SSRP1 R54A mutant, in which R54 was mutated to alanine (A). Radiometric metabolite–protein interaction assay was performed with ^14^C‐labeled pyruvate and purified recombinant GST‐SSRP1 WT or R54A, showing that, compared to SSRP1 wildtype (WT), SSRP1 R54A had much lower affinity for pyruvate (Figure 4D). Taken together, these results indicate that pyruvate directly interacts with SSRP1 at R54.

### 2.6 Pyruvate–SSRP1 Interaction Is Required for FACT‐Mediated *γ*H2AX Loading to Chromatin

To investigate the role of the interaction between pyruvate and SSRP1 in the regulation of *γ*H2AX levels in chromatin, we first examined the interaction between SSRP1 and *γ*H2AX in the presence of pyruvate and found that R54A mutation abrogated pyruvate‐increased interaction between SSRP1 and *γ*H2AX in vitro, as determined by a pulldown assay (Figure 4E). Moreover, in cells, the etoposide‐induced interaction between SSRP1 and *γ*H2AX in nonchromatin bound fraction was also attenuated by R54A mutation (Figure 4F). Of note, R54A did not influence the interaction between SSRP1 and SPT16, suggesting that R54A specifically disrupts the interaction between SSRP1 and *γ*H2AX without impairing the formation of FACT complex (Figure S4B, Supporting Information).

As reported recently, the FACT complex is the major regulator involved in H2AX exchange process that is modulated by H2AX phosphorylation.^[^ ^27^ ^]^ We thus asked whether pyruvate regulates *γ*H2AX levels in chromatin by FACT‐mediated *γ*H2AX exchange. To test this hypothesis, we carried out an in vitro histone exchange assay, in which H2AX‐H2B heterodimer was first phosphorylated by purified recombinant ATM (Figure S4C, Supporting Information), the serine/threonine–protein kinase that phosphorylates H2AX S139 at DSBs, and then mixed with commercial nucleosome, purified recombinant SPT16, SSRP1 WT or R54A, and pyruvate (Figure S4D, Supporting Information). Immunoblotting analyses of the nucleosome after the exchange assay showed that pyruvate enhanced FACT‐mediated *γ*H2AX loading to the nucleosome, while R54A mutation abrogated such FACT‐dependent *γ*H2AX loading (Figure 4G). Consistently, we depleted SSRP1 in U251 or U87 cells and rescued these cells with shRNA‐resistant (r) SSRP1 WT or R54A, the cells expressing rSSRP1 R54A had much less *γ*H2AX in chromatin than the cells expressing rSSRP1 WT after etoposide treatment (Figure 4H and Figure S4E,F, Supporting Information). Together, these results strongly suggest that pyruvate binds to SSRP1, which promotes FACT‐mediated *γ*H2AX loading to chromatin.

### 2.7 Pyruvate–SSRP1 Interaction Is Required for Tumor Cell Survival upon DNA Damage and Irradiation Resistance of Glioblastoma

**Figure 5:**
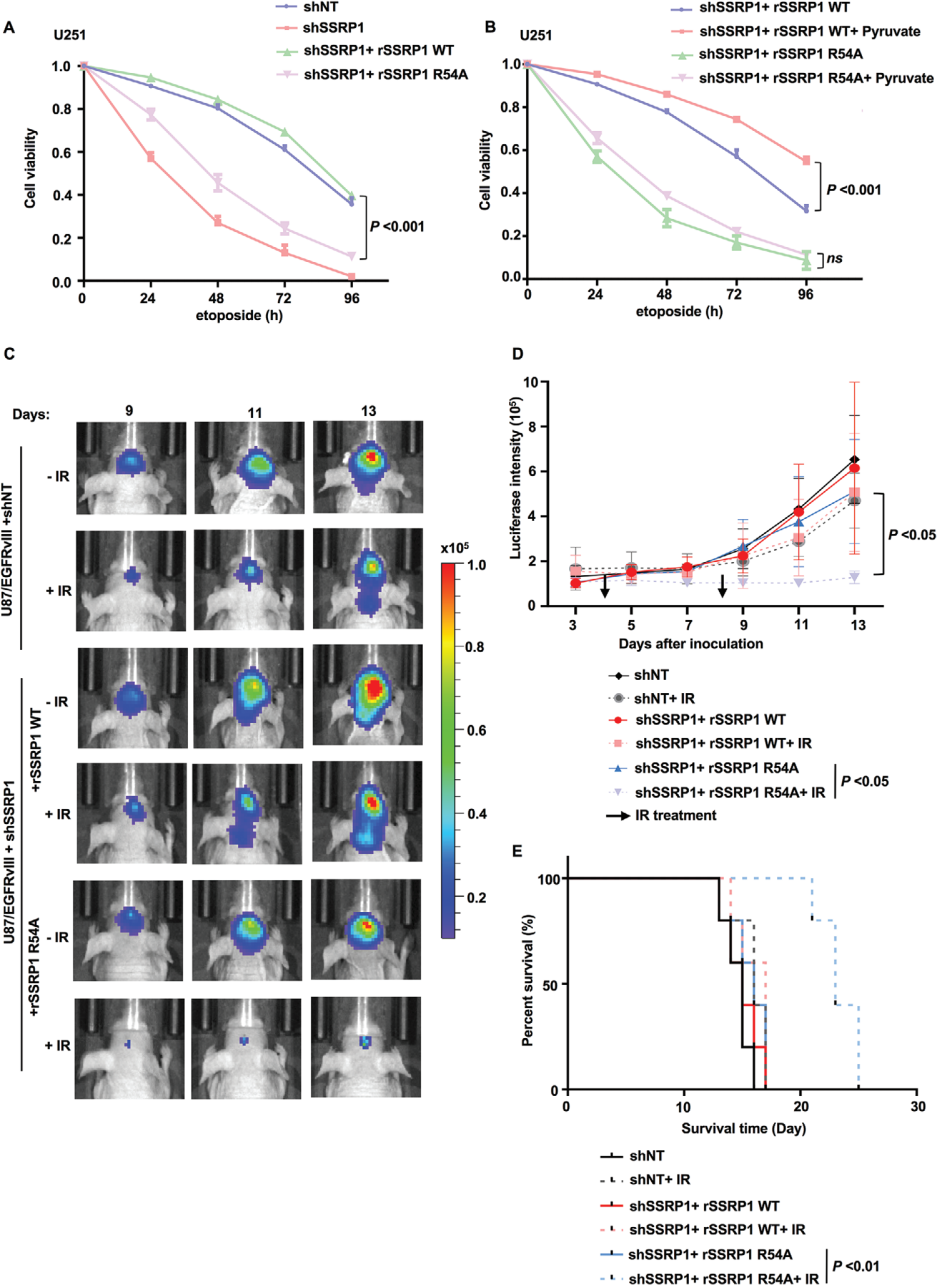
The binding of pyruvate to SSRP1 is required for tumor cell survival and irradiation resistance of glioblastoma. Data are representative of at least three independent experiments. A) SSRP1‐depleted U251 cells reconstituted with rSSRP1 WT or R54A were treated with etoposide (200 × 10^–6^ m) for indicated time. Cell viability was determined. Data represent the mean ± SD of the viability of the cells from three independent experiments (two‐tailed Student's t‐test). *P* values for comparisons between shSSRP1+rSSRP1 WT and shSSRP1+rSSRP1 R54A are shown. B) SSRP1‐depleted U251 cells reconstituted with rSSRP1 WT or R54A were supplemented with or without pyruvate (10 × 10^–3^ m, 3 h) and then treated with etoposide (200 × 10^–6^ m) for indicated time. Cell viability was determined. Data represent the mean ± SD of the viability of the cells from three independent experiments (two‐tailed Student's t‐test). C–E) U87/EGFRvIII cells stably expressing luciferase were infected with the lentivirus expressing shNT or shSSRP1 and the SSRP1‐depleted U87/EGFRvIII cells were reconstituted with the expression of rSSRP1 WT or R54A. These genetically modified cells (2 × 10^5^ per mouse) were intracranially injected into randomized athymic nude mice (five mice per group) and then treated with or without IR (X‐ray) radiation (6 Gy). Bioluminescence imaging of tumor growth was carried out. C) Representative real‐time images were presented and D) the intensities of luciferase were quantified using living image software (PerkinElmer). Data represent the mean ± SD of luciferase intensity of five mice per group (two‐tailed Student's t‐test). E) Survival durations of these implanted mice were compared (Log‐rank test).

To investigate the role of pyruvate–SSRP1 interaction in tumor cell survival, we depleted SSRP1 in U251 or U87 cells and rescued these cells with rSSRP1 WT or R54A. SSRP1 depletion markedly decreased the viability of tumor cells after etoposide treatment, while rescued expression of rSSRP1 WT completely recovered the viability of tumor cells to that of shNT‐expressing cells, excluding the off‐targeting possibility of shSSRP1. More importantly, compared to that of rSSRP1 WT, rescued expression of rSSRP1 R54A only slightly recovered the viability of SSRP1‐depleted tumor cells after etoposide treatment (**Figure** **5**A and Figure S5A, Supporting Information). Similar result was also obtained in the long‐term colony formation experiments (Figure S5C, Supporting Information), suggesting that the association of SSRP1 with pyruvate is required for tumor cell survival upon DNA damage.

To determine whether pyruvate supplementation promotes tumor cell survival upon DNA damage through its binding to SSRP1, we supplemented SSRP1‐depleted U251 or U87 cells rescued with rSSRP1 WT or pyruvate‐binding deficient mutant R54A with or without pyruvate, followed by the treatment of etoposide. The results showed that the expression of rSSRP1 R54A dramatically attenuated pyruvate‐enhanced tumor cell viability after etoposide treatment (Figure 5B and Figure S5B, Supporting Information), which confirms that the association of pyruvate with SSRP1 is also required for pyruvate supplementation‐enhanced tumor cell survival upon DNA damage.

We further confirmed the role of pyruvate‐regulated *γ*H2AX in irradiation resistance of glioblastoma. We intracranially injected SSRP1‐depleted U87 cells stably expressing EGFRvIII (U87/EGFRvIII) with or without rescued expression of rSSRP1 WT or R54A into randomized athymic nude mice, followed by the treatment of ionizing radiation (IR) (Figure S5D, Supporting Information). Bioluminescence imaging of mice showed that the tumors in the mice implanted with the cells expressing rSSRP1 R54A were more sensitive to IR treatment than the tumors in the mice implanted with the cells expressing rSSRP1 WT (Figure 5C,D). Moreover, those mice bearing with the tumors expressing rSSRP1 R54A had much longer survival duration than the mice bearing with the tumors expressing rSSRP1 WT after IR treatment (Figure 5E).

Collectively, these results demonstrate that the binding of pyruvate to SSRP1 promotes tumor cell survival and glioma radiation resistance.

### 2.8 AKT1‐Dependent PKM2 S222 Phosphorylation Is Necessary for PKM2 and FACT Interaction upon DNA Damage

**Figure 6:**
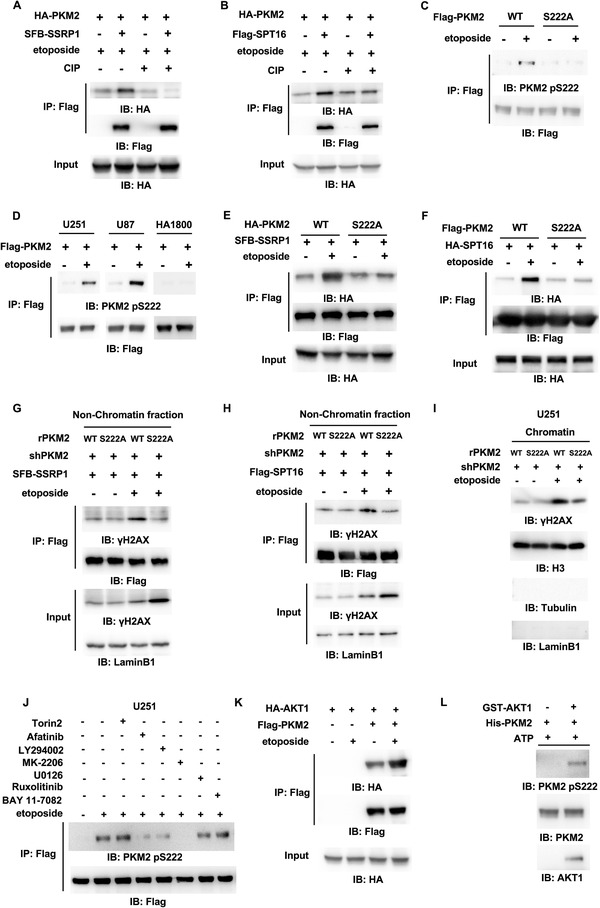
AKT1‐dependent PKM2 S222 phosphorylation is necessary for PKM2 and FACT interaction upon DNA damage. IP and IB analyses were performed with indicated antibodies. Data are representative of at least three independent experiments. A,B) Co‐IP was performed with anti‐Flag antibody in HEK293T cells transfected with A) SFB‐SSRP1 or B) Flag‐SPT16 and HA‐PKM2 after etoposide treatment (200 × 10^–6^ m, 1 h). The precipitated complex of SSRP1 or SPT16 was treated with or without CIP. C) HEK293T cells were transfected with Flag‐PKM2 WT or S222A and then treated with or without etoposide (200 × 10^–6^ m, 1 h). D) U251, U87 cells, or normal human astrocyte cells (HA1800) stably expressing Flag‐PKM2 were treated with or without etoposide (200 × 10^–6^ m, 1 h). E) HEK293T cells were transfected with SFB‐SSRP1 and HA‐PKM2 WT or S222A and then treated with or without etoposide (200 × 10^–6^ m, 1 h). F) HEK293T cells were transfected with HA‐SPT16 and Flag‐PKM2 WT or S222A and then treated with or without etoposide (200 × 10^–6^ m, 1 h). G,H) PKM2‐depleted HEK293T cells were rescued with rPKM2 WT or S222A. The cells were transfected with G) SFB‐SSRP1 or H) Flag‐SPT16 and then treated with or without etoposide (200 × 10^–6^ m, 1 h). Co‐IP experiment was performed with anti‐Flag antibody in the nonchromatin‐bound protein fractions. I) PKM2‐depleted U251 cells were rescued with rPKM2 WT or S222A and then treated with or without etoposide (200 × 10^–6^ m, 1 h). Chromatin fraction was prepared. J) U251 cells stably expressing Flag‐PKM2 were treated with or without ATM/DNA‐PK inhibitor Torin2 (100 × 10^–9^ m), EGFR inhibitor afatinib (5 × 10^–6^ m), PI3K inhibitor LY294002 (1 × 10^–6^ m), AKT1/2/3 inhibitor MK‐2206 (1 × 10^–6^ m), MEK1/2 inhibitor U0126 (5 × 10^–6^ m), JAK1/2 inhibitor ruxolitinib (50 × 10^–9^ m) or NF‐*κ*B inhibitor BAY11‐7082 (50 × 10^–6^ m) for 12 h and then treated with or without etoposide (200 × 10^–6^ m) for 1 h. K) Co‐IP was performed with anti‐Flag antibody in HEK293T cells transfected with HA‐AKT1 and Flag‐PKM2 after etoposide treatment (200 × 10^–6^ m, 1 h). L) In vitro kinase assays were carried out with purified recombinant His‐PKM2 and commercially purchased recombinant GST‐AKT1.

The protein–protein interaction is frequently regulated by posttranslational modification (PTM). Phosphorylation is one of the most prevalent PTM in mammalian cells.^[^ ^28^ ^]^ To explore the mechanism underlying the interaction between PKM2 and FACT upon DNA damage, we asked whether phosphorylation is required for their interaction. The immunoprecipitants of SFB‐SSRP1 or Flag‐SPT16 were treated with nonspecific calf‐intestinal alkaline phosphatase (CIP), which showed that CIP treatment disrupted the interaction between PKM2 and the FACT complex (**Figure** **6**A,B), confirming that the interaction of these two proteins is phosphorylation dependent.

To identify the phosphorylated residues in PKM2, we carried out mass spectrometry analyses of immunoprecipitated PKM2 and observed that S222 was identified as the most possible residue phosphorylated after etoposide treatment (Figure S6A,B, Supporting Information). A custom‐designed antibody specifically against phosphorylated PKM2 S222 (PKM2 pS222) was generated. Immunoblotting analysis with this anti‐PKM2 pS222 antibody showed that PKM2 was indeed phosphorylated at S222 after etoposide treatment, while S222A mutation completely abrogated such phosphorylation (Figure 6C), validating the specificity of this antibody. Notably, PKM2 pS222 was generally detected in U251 and U87 cells, but not in normal human astrocyte cells (HA1800) after etoposide treatment (Figure 6D), suggesting that PKM2 pS222 is specifically induced in tumor cells.

To confirm whether PKM2 pS222 is required for the interaction between PKM2 and FACT, co‐IP experiment was performed, which showed that S222A mutation abrogated etoposide‐induced interaction between PKM2 and SSRP1 or SPT16 (Figure 6E,F), without altering the enzymatic activity of PKM2 (Figure S6C, Supporting Information). Due to losing interaction with PKM2, the FACT complex had much less interaction with nonchromatin‐bound *γ*H2AX in PKM2‐depleted cells rescued with rPKM2 S222A than in the cells rescued with rPKM2 WT after etoposide treatment (Figure 6G,H and Figure S6D, Supporting Information). And, consequently, less *γ*H2AX in chromatin was detected in PKM2‐depleted U251 cells rescued with rPKM2 S222A than in the cells rescued with rPKM2 WT (Figure 6I).

To identify the kinase that phosphorylates PKM2 pS222, we pretreated U251 cells with a panel of inhibitors of multiple signal pathways reported to be activated upon DNA damage, such as ATM/DNA‐PK inhibitor (Torin2) and the inhibitors of EGFR signaling frequently activated in glioblastoma, including EGFR inhibitor (Afatinib), PI3K inhibitor (LY294002), AKT1/2/3 inhibitor (MK‐2206), MEK1/2 inhibitor (U0126), JAK1/2 inhibitor (Ruxolitinib), NF‐*κ*B inhibitor (BAY11‐7082), followed by the treatment of etoposide. Immunoblotting analyses with anti‐PKM2 pS222 showed that inhibiting EGFR, PI3K or AKT blocked etoposide‐induced PKM2 pS222 (Figure 6J and Figure S6E, Supporting Information). Co‐IP experiments showed that the association of PKM2 with AKT1, but not with AKT2, AKT3, or EGFR, was enhanced by etoposide treatment (Figure 6K and Figure S6F–H, Supporting Information). Of note, etoposide treatment did not affect the activation of AKT (Figure S6I, Supporting Information). Furthermore, we performed an in vitro kinase assay by mixing commercial purchased GST‐AKT1 and purified recombinant PKM2, which confirmed that AKT1 directly phosphorylated PKM2 at S222 (Figure 6L).

Taken together, these results demonstrate that AKT1 interacts with PKM2 and phosphorylates PKM2 S222 upon DNA damage, which is required for the interaction between PKM2 and FACT, the interaction between FACT and nonchromatin‐bound *γ*H2AX and subsequent *γ*H2AX loading to chromatin.

### 2.9 PKM2 pS222 Is Required for DNA Repair, Tumor Cell Survival and Radiation Resistance of Glioblastoma

**Figure 7:**
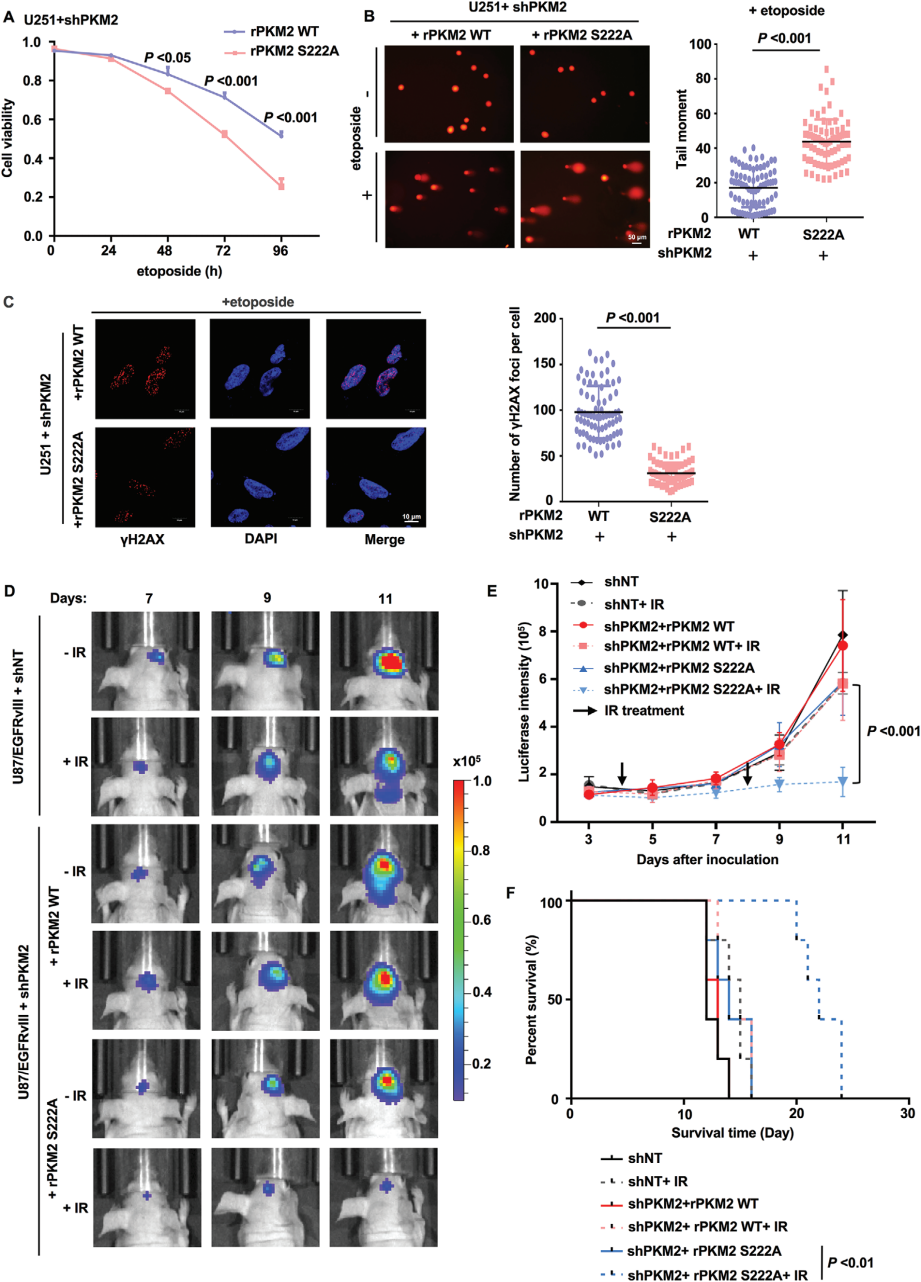
PKM2 pS222 is required for DNA repair, tumor cell survival, and radiation resistance of glioblastoma. A–C) PKM2‐depleted U251 cells were rescued with rPKM2 WT or S222A. A) Cells were treated with etoposide (200 × 10^–6^ m) for indicated time. Cell viability was determined. Data represent the means ± SD of the viability of the cells from three independent experiments (two‐tailed Student's t‐test). B) The cells were treated with or without etoposide (500 × 10^–6^ m, 3 h). The representative images of the comet assay in these cells were shown (left panel). Scatter dot plot (right panel) of the tail moments in the comet assay from shPKM2+rPKM2 WT cells (*n* = 80) or shPKM2+rPKM2 S222A cells (*n* = 80) treated with etoposide. Data represent mean ± SD of the tail moments (Mann Whitney test). Data are representative of three independent experiments. C) The cells were treated with etoposide (40 × 10^–6^ m, 0.5 h). IF staining was performed using anti‐*γ*H2AX antibody. Representative images were shown (left panel). Scatter dot plot (right panel) of the number of *γ*H2AX foci per cell. Data represent the mean ± SD of the number of *γ*H2AX foci from shPKM2+rPKM2 WT cells (*n* = 76) and shPKM2+rPKM2 S222A cells (*n* = 72) treated with etoposide (Mann Whitney test). Data are representative of three independent experiments. D–F) U87/EGFRvIII cells stably expressing luciferase were infected with the lentivirus expressing shNT or shPKM2 and the PKM2‐depleted U87/EGFRvIII cells were reconstituted with the expression of rPKM2 WT or S222A. These genetically modified cells (2 × 10^5^ per mouse) were intracranially injected into randomized athymic nude mice (five mice per group) and then treated with or without IR (X‐ray) radiation (6 Gy). D) Bioluminescence imaging of tumor growth was carried out. E) Representative real‐time images were presented (left panel) and the intensities of luciferase were quantified (right panel) using living image software (PerkinElmer). F) Data represent the mean ± SD of luciferase intensity of five mice per group (two‐tailed Student's t‐test). Survival durations of these implanted mice were compared (Log‐rank test).

To investigate the role of PKM2 pS222 in DNA repair, we treated PKM2‐depleted U251 or U87 cells rescued with rPKM2 WT or S222A with etoposide (Figures S6D and S7A, Supporting Information). Cell viability assay and the long‐term colony formation assay showed that the cells rescued with rPKM2 S222A were more sensitive to etoposide than the cells rescued with rPKM2 WT (**Figure** **7**A and Figure S7B,C, Supporting Information). The comet assay showed that more DNA damage was observed in tumor cells rescued with rPKM2 S222A than in the cells rescued with rPKM2 WT after etoposide treatment (Figure 7B and Figure S7D, Supporting Information). IF staining with anti‐*γ*H2AX or anti‐MDC1 antibody showed that tumor cells rescued with rPKM2 S222A had much less *γ*H2AX and MDC1 foci than the cells rescued with rPKM2 WT (Figure 7C and Figure S7E, Supporting Information) after etoposide treatment.

We next investigated the role of PKM2 pS222 in glioma radiation resistance. We generated PKM2‐depleted U87/EGFRvIII cells rescued with or without rPKM2 WT or S222A (Figure S7F, Supporting Information) and intracranially injected these cells into randomized nude mice, followed by the IR treatment. Bioluminescence imaging of mice showed that the tumors in the mice implanted with the cells expressing rPKM2 S222A were more sensitive to IR treatment than the tumors in the mice implanted with the cells expressing rPKM2 WT (Figure 7D,E). Similarly, the mice with the tumors expressing rPKM2 S222A had much longer survival time than the mice with the tumors expressing rPKM2 WT after IR treatment (Figure 7F).

Collectively, these results demonstrate that PKM2 pS222 is essential for DNA repair, tumor cell survival, and radiation resistance of glioblastoma.

### 2.10 PKM2 pS222 Levels Correlate with the Malignancy and Prognosis of Human Glioblastoma

**Figure 8:**
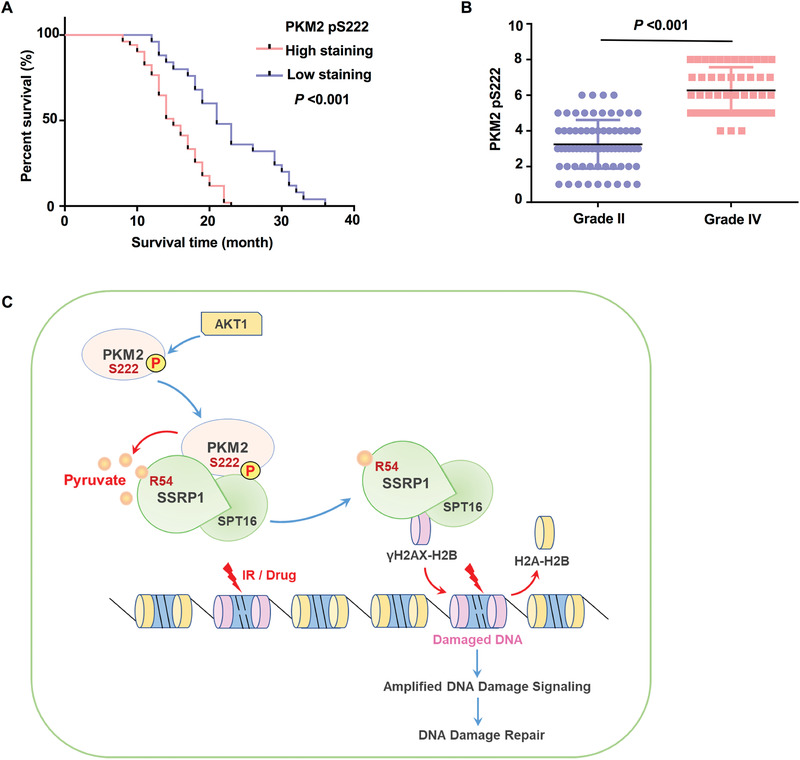
PKM2 pS222 levels correlate with the malignancy and prognosis of human glioblastoma. A) Survival of 76 patients with low (0–4.9 staining scores, blue curve) versus high (5.0–8 staining scores, red curve) PKM2 S222 phosphorylation levels (low, 25 patients; high, 51 patients) was compared (Log‐rank test). B) 73 diffuse astrocytoma (Grade II) specimens were immunohistochemically stained using anti‐PKM2 pS222 antibody. Staining scores of the specimens were compared with 48 stained GBM (Grade IV) specimens (two‐tailed Student's t‐test). C) Upon DNA damage, PKM2 is phosphorylated by AKT1 at serine (S) 222. Phosphorylated PKM2 interacts with the FACT complex and provides a local source of pyruvate. Pyruvate physically binds to SSRP1 and enhances the association of the FACT complex and *γ*H2AX, which facilitates FACT‐mediated chromatin loading of *γ*H2AX, thereby amplifying DNA damage signaling and promoting DNA damage repair.

To define the clinical relevance of PKM2 S222 phosphorylation, we performed immunohistochemistry (IHC) analyses in the tumor tissues from human primary glioblastoma multiforme (GBM) patients using anti‐PKM2 pS222 antibody. The antibody specificity was validated by IHC analyses of GBM specimens with specific blocking peptides (Figure S8, Supporting Information). The survival durations for the 76 GBM patients, all of whom received standard adjuvant radiotherapy after surgery, followed by treatment with an alkylating agent (temozolomide in the majority of cases), were compared between the patients with low PKM2 pS222 (0–4.9 staining) and the patients with high PKM2 pS222 (5–8 staining). Patients (25 cases) whose tumors had low levels of PKM2 pS222 had a median survival of 21 months, and patients (51 cases) whose tumors had high levels of PKM2 pS222 had a significantly lower median survival of 15 months (**Figure** **8**A).

In addition, we determined the relationship between PKM2 pS222 levels and the grades of glioma. PKM2 pS222 levels in the tumors from patients (73 cases) with low‐grade diffuse astrocytoma (WHO grade II; median survival time >5 years) were compared with those in the tumors from patients (48 cases) with high‐grade GBM. IHC analyses showed that lower PKM2 pS222 levels were observed in low‐grade tumors than in high‐grade GBM specimens (Figure 8B).

Collectively, these results support a role for PKM2 pS222 in the clinical behavior of human GBM and reveal a relationship between PKM2 pS222 and the prognosis of the tumor.

## 3 Discussion

IR and most of the chemotherapeutic agents, as the primary antitumor therapies, induce cell death by directly or indirectly causing DNA damage. Deregulated DNA repair mechanisms may contribute to hypersensitivity or resistance of cancer cells to genotoxic agents. Targeting DNA repair pathway can increase the tumor sensitivity to cancer therapies. Enhanced glycolysis in tumor cells has been recently established to be responsible for the radiation resistance of tumors, partly by enhancing DNA repair. However, the mechanism underlying how glycolysis, especially the glycolytic metabolite, regulates DNA repair in tumor cells remains largely unknown. In this study, we identified pyruvate, a key glycolytic metabolite produced by PKM2, as a signal molecule to intensify DNA damage signaling by facilitating FACT‐mediated *γ*H2AX loading to chromatin, thereby promoting tumor cell survival upon DNA damage (Figure 8C). This finding reveals a new role of pyruvate in DNA repair and glioma radiation resistance, which not only link cell metabolism to DNA repair but also implicates a new strategy to improve the efficacy of existing therapies for glioblastoma.

Aerobic glycolysis provides energy and building blocks for biosynthesis, thereby conferring a growth advantage on tumor cells.^[^ ^29^ ^]^ Given that aerobic glycolysis is a distinctive hallmark of cancer, antitumor therapeutic agents targeting aerobic glycolysis are being developed.^[^ ^30^ ^]^ Emerging evidence demonstrates that enhanced glycolysis in tumor cells may not only supports cell growth but also maintains cell immortality and drives therapeutic resistance at least partly by promoting DNA repair.^[^ ^8^, ^31^ ^]^ However, it remains unclear how deregulated glycolysis in tumor cells is involved in the repair of DNA damage. In the present study, we show that the rate‐limiting glycolytic enzyme PKM2 interacts with a histone chaperon FACT and provides a local source of pyruvate which directly binds to and facilitates FACT‐mediated *γ*H2AX loading to chromatin, thereby promoting the repair of DNA damage in tumor cells. Notably, etoposide treatment did not change the glycolytic activity and the levels of total pyruvate in tumor cells (Figure S3C, Supporting Information), indicating that the new function of glycolytic enzyme PKM2, instead of deregulated glycolysis, promotes DNA repair of tumor cells.

Pyruvate, normally derived from glucose through glycolysis, is the simplest *α*‐keto acid with a carboxylic acid and a ketone functional group, which unites several key metabolic processes. It can not only be converted back to carbohydrates (such as glucose) via gluconeogenesis, or to fatty acids from Acetyl‐CoA, but also be used to synthesize the amino acid alanine, or be metabolized into ethanol or lactic acid via fermentation.^[^ ^32^ ^]^ Interestingly, pyruvate has been recently shown to regulate genome‐wide acetylation of histone proteins. In response to growth signal, pyruvate dehydrogenase (PDH) complex is translocated to the nucleus and converts pyruvate to acetyl‐CoA for the histone acetylation important for G1‐S transition and the expression of S phase markers.^[^ ^33^ ^]^ Interestingly, it has also been shown that pyruvate reduces hypoxia and reoxygenation‐induced DNA damage, probably due to that pyruvate restores glutathione levels during reoxygenation.^[^ ^34^ ^]^ Glutathione is an important intracellular antioxidant that plays a vital role in cellular protection against damage by free radicals, peroxides, and toxins. In our study, however, we demonstrate that pyruvate directly enhances DNA repair by binding to and facilitating FACT‐mediated chromatin loading of *γ*H2AX, thereby promoting tumor cell survival upon chemo drug and ionizing radiation‐induced DNA damage. Unlike the detoxification of ROS, pyruvate has been demonstrated in our study to directly regulate DNA repair signaling through a histone chaperon FACT.

Intriguingly, exogenous pyruvate sufficiently promotes DNA repair and tumor cell survival upon DNA damage. Given that tumor cells can export pyruvate to microenvironment via monocarboxylate transporter 1 (MCT1), we have reason to believe that tumor cells with high levels of pyruvate could promote the resistance of neighbor tumor cells to DNA damage by the paracrine mechanism, thereby implicating the therapeutic opportunity of lowering intracellular pyruvate levels by pharmacological inhibition to increase the efficacy of current therapeutics for glioblastoma.

The FACT complex is involved in multiple processes, such as mRNA elongation, DNA replication, and DNA repair. For instance, it destabilizes and restores nucleosomal structure as a histone chaperone during transcription elongation.^[^ ^22^, ^35^ ^]^ Upon UV irradiation, the FACT complex associates with casein kinase 2 (CK2) and promotes its phosphorylation and activation of p53.^[^ ^21^ ^]^ As shown in an in vitro histone exchange assay, both H2AX incorporation into and H2AX dissociation from the nucleosome is facilitated by the FACT complex. In addition, the phosphorylation of nucleosomal H2AX by DNA‐PK facilitates FACT‐induced dissociation of H2AX, while ADP‐ribosylation of SPT16 promotes the dissociation of FACT from the nucleosome, which leads to the stabilization of nucleosomal H2AX.^[^ ^27^ ^]^ But whether this proposed mechanism exactly exists in tumor cells treated with DNA‐damage agents remains unclear. A most recent study shows that FACT promotes the deposition of newly synthesized H2AX at sites of DNA synthesis during DNA replication and repair.^[^ ^36^ ^]^ However, it remains unclear how the function of the FACT complex is regulated upon DNA damage, especially by cell metabolism. In this study, we reveal for the first time that the function of the FACT complex is regulated by a glycolytic metabolite, pyruvate which promotes FACT‐mediated chromatin loading of *γ*H2AX, thereby promoting DNA repair in tumor cells.

As a rate‐limiting glycolytic enzyme, PKM2 plays a central role in tumor cell proliferation. However, recently, rapidly accumulating evidence indicates that far beyond its metabolic functions, PKM2 performs multiple nonmetabolic functions, including protein kinase and transcriptional coactivator, to regulate tumor cell proliferation, survival, and migration.^[^ ^11^, ^16^, ^37^, ^38^ ^]^ For example, under oxidative stress, PKM2 is translocated to mitochondria and phosphorylates antiapoptotic protein Bcl2 to prevent its proteasome‐mediated degradation, thereby inhibiting oxidative stress‐induced tumor cell apoptosis.^[^ ^39^ ^]^ More recently, Sizemore et al. demonstrate that in response to IR or oxidative stress, PKM2 phosphorylates CtIP to increase its recruitment at DSBs and resection of DNA ends, thereby promoting homologous recombination (HR)‐mediated DNA DSB repair.^[^ ^40^ ^]^ In this study, we discovered another critical mechanism underlying PKM2‐promoted DNA repair, in which PKM2 regulates FACT‐mediated *γ*H2AX loading to chromatin through providing a local source of pyruvate, thereby amplifying DNA damage response.

Notably, it has also been demonstrated that moderate DNA damage induced by low dose of etoposide promotes the metabolism of phosphate pentose pathway by increasing PKM2 Y105 phosphorylation, which favors the survival of tumor cells.^[^ ^41^ ^]^ In our study, however, we did not detect the phosphorylation of Y105 in our mass spectrometry experiment, suggesting that Y105 is not abundantly phosphorylated in our context. Moreover, the treatment of etoposide did not influence the glycolytic activity of tumor cells we used. From our perspective, the difference in the observations of different phosphorylation sites is mainly and very likely due to the different cell lines and etoposide dosage used in these two studies. However, beyond cell type and etoposide dosage, we do notice that they detected the phosphorylation of PKM2 Y105 at 24 h after etoposide treatment. In contrast, we observed S222 phosphorylation at 1 h after etoposide treatment. Due to the nature of highly dynamic regulation of phosphorylation, our proposed mechanism may work in the early stage of DNA damage response.

In addition, we found that PKM2‐regulated DNA repair requires the phosphorylation of PKM2 S222, which increases the interaction between PKM2 and the FACT complex, thereby promoting FACT‐mediated *γ*H2AX loading to chromatin. Importantly, IHC analyses of human glioblastoma with anti‐PKM2 pS222 antibody suggest that PKM2 pS222 levels have the strong correlation with the malignancy and prognosis of human glioblastoma, highlighting the prognostic potential of PKM2 pS222 levels for glioblastoma.

## 4 Experimental Section

### Materials

*Antibodies*: Mouse monoclonal antibodies against Tubulin (1:5000, T5201), Flag (1:5000, F3165) and anti‐Flag M2 affinity gel (A2220) were purchased from Sigma (St. Louis, MO, USA). Rabbit monoclonal antibodies against PKM2 (1:3000, 4053S), HA (1:3000, 3724S), *β*‐actin (1:3000, 3700S), LaminB1 (1:3000, 12586s), H2AX pS139 (1:3000, 9718S), ATM pS1981 (1:1000, 13050S), ATM (1:1000, 2873), AKT pT308 (1:1000, 4056S), and rabbit polyclonal antibody against EGFR pY1068 (1:1000, 2234S) were obtained from Cell Signaling Technology (Danvers, MA, USA). Rabbit polyclonal antibody against histone H3 (1:2000, ab1791) were purchased from Abcam (Cambridge, USA). Mouse monoclonal antibody against GST (1:2000, sc‐138), SSRP1(1:1000, sc‐74536), p‐ERK (1:1000, sc7383), and rabbit polyclonal antibody against ERK1 (1:1000, sc94) were purchased from Santa Cruz Biotechnology (Santa Cruz, CA, USA). Rabbit polyclonal antibody against H2AX (1:2000, A11361), MDC1 (1:500, A8358), AKT1 (1:1000, A11016), EGFR (1:1000, A3598), AKT1 pS473 (1:1000, AP0140) and the custom‐designed rabbit polyclonal antibody against phospho‐PKM2 S222 [C‐DLPAV(S‐p) EKD] (1:1000 for IB, 1:100 for IHC) were obtained from Abclonal Technology (Wuhan, China).

*Reagents*: Puromycin (540411‐100MGCN), hygromycin (400052‐20MLCN), and human recombinant histone H2AX‐H2B dimers (14‐1055) were bought from Merck/Millipore (Darmstadt, Germany). Pyruvate (sodium salt) (P5280), NADH (N8129), and ADP (A2754) were purchased from Sigma (St. Louis, MO, USA). ATP (tlrlatp) was purchased from InvivoGen (CA, USA). DNA transfection reagent Hieff Trans Liposomal Transfection Reagent (H17520) was purchased from Yeasen Biotechnology (Shanghai, China). Recombinant mononucleosomes‐biotinylated (31467) were bought from Active Motif (Carlsbad, USA). Recombinant GST‐AKT1 (A16‐10G) was bought from SignalChem (British Columbia, Canada). The ATM inhibitor (Torin2), EGFR inhibitor (Afatinib), MEK inhibitor (U0126), AKT inhibitor (MK‐2206), PI3K inhibitor (LY294002), JAK1/2 inhibitor (Ruxolitinib), NF‐*κ*B inhibitor (BAY11‐7082), and PKM2 inhibitor (Shikonin) were obtained from Selleck.

### Plasmids

PCR‐amplified PKM2 was cloned into pCMV‐Flag, pCDH‐Flag, pCDNA3‐HA, pLVX‐IRES‐ZsGreen‐newMCS, and pCold I‐His. PCR‐amplified SSRP1 or SPT16 was cloned into pCDNA3.0‐HA, pCMV‐Flag, pCDH‐SFB, pGEX KG, and pET28as. PKM2 and SSRP1 mutations were generated using the QuickChange site‐directed mutagenesis kit (Stratagene, La Jolla, CA, USA). The pGIPZ control was generated with the control oligonucleotide 5ʹ‐CTCGCTTGGGCGAGAGTAA‐3ʹ. pGIPZ PKM2 shRNA was generated with 5′‐CATCTACCACTTGCAATTA‐3′ oligonucleotide targeting exon 10 of the PKM2 transcript. pGIPZ SSRP1 shRNA was generated with 5ʹ‐ GGCAAGACCTTTGACTACA‐3ʹ oligonucleotide targeting the coding region of the SSRP1 transcript. rPKM2 contains same sense mutations of C1170T, C1173T, T1174C, and G1176T. rSSRP1 contains nonsense mutations of C681T, G684A, C687A, T690C, C693T.

### Cell Culture and Transfection

U87, U251 GBM cells, HA1800, and HEK293T cells were obtained from the cell library of the Chinese Academy of Sciences and maintained in high glucose (sodium pyruvate‐free) Dulbecco's modified Eagle's medium (DMEM) supplemented with 10% fetal bovine serum (FBS). The protein expression and reconstitution experiments were conducted using the established stable cell lines. Cells were transfected with indicated plasmids using Liposomal Transfection Reagent according to the manufacturer's instructions.

### Cell Viability Assay

In total, 2 × 10^5^ cells were seeded in six‐well plates. After etoposide treatment for the indicated time, cells were harvested with trypsin. Cells were stained with Trypan blue and cell viability was measured by Thermo Countess II FL.

### Clonogenic Survival Assays

Survival curves in clonogenic assays were analyzed as previously described in ref. [42] with minor modification. In short, the cells were seeded into six‐well plates, and Etoposide was added after the cells were attached, then cultured for 1–2 weeks until visible colonies formed. The colonies were washed with PBS and stained with 0.1% crystal violet for 20 min.

### Comet Assay

Cells were seeded in six‐well plates and then treated with or without etoposide (500 × 10^–6^ m) for 3 h before harvesting by trypsinization. Cells were resuspended in PBS at a concentration of approximately 500 cells µL^–1^. 10 µL cell suspension was mixed with 85 µL 0.6% low melting agarose at 37 ℃. The mixture was added to prewarmed (37 ℃) agarose coated fully frosted slides (Thermo‐Fisher Scientific) and a coverslip was added on top of the mixture. The slides were kept on ice for 10 min before removing the coverslip and incubated in lysis buffer (10 × 10^–3^ m Tris pH 10, 2.5 m NaCl, 0.1 m EDTA, 10% DMSO, and 1% Triton X‐100) at 4 ℃ for 3 h in the dark. Slides were washed with PBS three times and incubated in alkaline electrophoresis buffer (0.3 n NaOH, 1 × 10^–3^ m EDTA) for 30 min. Electrophoresis was run at 300 mA, 25 V for 30 min in electrophoresis buffer using a Comet Assay tank (Thistle Scientific). Slides were washed in neutralization buffer (0.4 m Tris‐HCl pH7.5) and counterstained with EB dye (Sangon Biotech). DNA damage was measured in terms of tail moments using CASP software.

### Immunofluorescence Analysis

Cells were fixed and incubated with primary antibodies, Alexa Fluor dye‐conjugated secondary antibodies, and DAPI according to standard protocols. Cell imaging was performed on a FLUOVIEW FV3000 laser scanning confocal microscope (OLYMPUS). *γ*H2AX and MDC1 foci were quantified using the Image‐Pro Plus software.

### Subcellular Fractionation

Chromatin‐bound fraction and nonchromatin‐bound fraction were obtained as previously described in refs. [43, 44, 45] with minor modification. Briefly, U251 or U87 cells were treated with or without etoposide (200 × 10^–6^ m) for 1 h. Cells were collected and resuspended in lysis buffer [10 × 10^–3^ m HEPES (pH 7.9), 10 × 10^–3^ m KCl, 1.5 × 10^–3^ m MgCl_2_, 0.5% NP‐40] with phosphatase inhibitors and protease inhibitors and incubated on ice for 20 min. The cytosolic fraction was separated from the nuclei by centrifugation at 16 000 *g* at 4 ℃ for 15 min. The nuclear pellet was washed and resuspended in low salt buffer [10 × 10^–3^ m Tris‐HCl (pH 7.6), 0.2 × 10^–3^ m MgCl2, 1% Triton X‐100] with phosphatase inhibitors and protease inhibitors, and incubated on ice for 2 h. The nonchromatin‐bound fraction was separated by centrifugation at 16 000 *g* at 4 ℃ for 15 min. Chromatin‐bound proteins were released from the DNA by addition of 0.2 n HCl and incubation on ice overnight, followed by centrifugation at 16 000 *g* at 4 ℃ for 15 min, the supernatant is the chromatin fraction.

### Glucose Consumption and Lactate Production

U251 cells were seeded in 12‐well plate and the media was replaced after cell attachment. 24 h later, the media were collected for measurement of glucose and lactate concentrations as determined by glucose assay kit (Sigma) and lactate assay kit (Biovision). Glucose consumption and lactate production were normalized by cell numbers.

### Pyruvate Assay

Pyruvate concentration was measured using Pyruvate Assay Kit (Sigma) according to the manufacturer's instructions. As for the determination of pyruvate content in cytoplasm and nucleus, cells were treated with etoposide for the indicated times and were collected and washed three times with cold PBS. Cells were then lysed in lysis buffer [10 × 10^–3^ m HEPES (pH 7.9), 10 × 10^–3^ m KCl, 1 × 10^–3^ m DTT, 0.1 × 10^–3^ m EDTA, 0.1 × 10^–3^ m EGTA, 0.5 × 10^–3^ m PMSF, 0.5% NP‐40] supplemented with phosphatase inhibitors and protease inhibitors. Following centrifugation at 1000 *g* for 5 min, the supernatants were collected as the cytoplasmic fraction. Pellets were washed twice with cold PBS and then lysed in Pyruvate Assay Buffer (Sigma) for pyruvate content detection.

### Mass Spectrometry Analysis

Immunoprecipitated Flag‐PKM2 using Flag beads (Sigma) from U251 cells was treated with or without Etoposide (200 × 10^–3^ m) for 1 h. The precipitated complexes were boiled at 95 ℃ for 10 min. Flag‐PKM2 for detection of PKM2 phosphorylation was separated from the complexes using SDS‐PAGE gel.

### In‐Gel Digestion

Briefly, gel band was destained with 50 × 10^–3^ m NH4HCO3 in 50% acetonitrile. The protein was reduced with 10 × 10^–3^ m TCEP (Thermo Scientific) for 30 min and alkylated with 55 × 10^–3^ m iodoacetamide (Sigma) in the dark for 30 min, respectively. 12.5 ng µL^–1^ trypsin (Promega) in 50 × 10^–3^ m NH4HCO_3_ was used for digestion overnight at 37 ℃. The digestion was stopped by and extracted with 50% acetonitrile/5% formic acid and dried out by Speed Vacuum instrument. The sample was reconstituted with 0.1% formic acid, then desalted using a Mono‐Spin C18 column (GL Science, Tokyo, Japan), and dried out by Speed Vacuum instrument.

### HPLC‐Tandem MS (MS/MS) Analysis of Peptides

The peptide mixture was analyzed by a homemade 15 cm long pulled‐tip analytical column (75 µm ID packed with Aqua C18‐3 µm resin, Phenomenex), the column was then placed in line with an Easy‐nLC 1200 nano HPLC (Thermo Scientific) for mass spectrometry analysis. The analytical column temperature was set at 55 ℃ during the experiments. The mobile phase was 0.1% formic acid in water as buffer A and 0.1% formic acid in 80% acetonitrile as buffer B, 0–1 min, 1%–4% B; 1–91 min, 4–35% B; 91–101 min, 35%–60% B, 101–111 min, 60%–100% B, 111–120 min, 100% B. The flow rate was set as 300 nL min^–1^.

Data‐dependent MS/MS analysis was performed with a Q Exactive Orbitrap mass spectrometer (Thermo Scientific). Peptides eluted from the LC column were directly electrosprayed into the mass spectrometer with the application of a distal 2.5‐kV spray voltage. A cycle of one full‐scan MS spectrum (m/z 300–1800) was acquired followed by top 20 MS/MS events at a 30% normalized collision energy. Full scan resolution was set to 70 000 with automated gain control (AGC) target of 3e6. MS/MS scan resolution was set to 17 500 with isolation window of 1.8 m/z and AGC target of 1e5. MS scan functions and LC solvent gradients were controlled by the Xcalibur data system (Thermo Scientific).

### Data Analysis

The acquired MS/MS data were analyzed against a human UniProtKB database (released on Aug‐12th 2019) using PEAKS Studio 8.5. Cysteine alkylation by iodoacetamide was specified as fixed modification with mass shift 57.02 and methionine oxidation as a variable modification with mass shift 15.99. Additionally, phosphorylation at S/T/Y was set as a variable modification with mass shift of 79.97. Trypsin was set as the cleavage enzyme, with both specific at peptide N/C term; the max missed cleavage was set as 3.

In order to accurately estimate peptide probabilities and false discovery rates, a decoy database containing the reversed sequences of all the proteins was appended to the target database to accurately estimate peptide probabilities and false discovery rate (FDR), the FDR was set at 0.01.

### Measurement of PKM2 Enzymatic Activity

PKM2 activity was measured by an LDH coupled assay. The reaction mixture contained 50 × 10^–3^ m Tris–HCl (pH 7.5), 100 × 10^–3^ m KCl, 10 × 10^–3^ m MgCl_2_, 0.6 × 10^–3^ m PEP, 0.9 × 10^–3^ m ADP, 0.12 × 10^–3^ m *β*‐NADH and 4.8 U mL^–1^ LDH. In the inhibition assay, 1 µL of Shikonin solution or DMSO solution was added to the 9 µL preincubated mixture. The total 10 µL mixture was incubated for 25 min at 25 °C before 125 µL of reaction solution was added to the mixture. PKM2 activity was calculated at 25 °C by monitoring change of NADH which has absorbance at 340 nm from 0 to 20 min.

### Gel Filtration

U87 cells stably expressing Flag‐PKM2 were treated with etoposide (200 × 10^–6^ m, 1 h). Nuclear fractions were prepared in these cells. Flag‐PKM2 was immunoprecipitated from nuclear fractions and eluted with flag peptide in TBS buffer (25 × 10^–3^ m Tris, 150 × 10^–3^ m NaCl, pH7.2). The gel filtration column (Superdex 200 increase 10/300 GL, Cytiva) was washed and equilibrated by cold TBS (4 °C). Nuclear PKM2 was passed over gel filtration column. The speed rate of flow is 0.5 mL min^–1^. Fractions were collected every 0.5 mL per tube and analyzed by western blot.

### Purification of Recombinant Proteins

WT and mutant GST‐SSRP1, His‐SSRP1, GST‐SPT16, and His‐PKM2 were expressed in bacteria and purified as described previously.^[^ ^46^ ^]^

### In Vitro Kinase Assay

In brief, purified monomeric ATM from Yanhui Xu lab (Shanghai Medical College of Fudan University, China) was incubated with commercial H2AX‐H2B in 40 µL kinase buffer (50 × 10^–3^ m HEPES pH 8.0, 50 × 10^–3^ m KCl, 10% glycerol, 5 × 10^–3^ m MgCl_2_, 2 × 10^–3^ m ATP and 1 × 10^–3^ m DTT) at 30 ℃ for 90 min. The AKT1 in vitro kinase assays were performed in buffer (20 × 10^–3^ m Tris‐HCl [pH 7.2], 2.5 × 10^–3^ m MnCl_2_, 2.5 × 10^–3^ m MgCl_2_, 0.5 × 10^–3^ m CaCl_2_, 1 × 10^–3^ m DTT, and 100 × 10^–6^ m ATP) at 30 ℃ for 1 h. Reactions were terminated by the addition of SDS‐PAGE sample loading buffer and analyzed by western blotting.

### Radiometric Metabolite–Protein Interaction Assays

Bead‐bound bacterial‐purified GST tagged SSRP1 WT or R54A was incubated with 0.12 µCi of ^14^C‐pyruvate for 30 min. Then the beads were washed and eluted. The radioactivity was detected by scintillation counting (PerkinElmer).

### Surface Plasmon Resonance (SPR)

The SPR analysis was performed using a Biacore8K instrument (GE Healthcare). Purified his‐SSRP1 protein was coupled to carboxymethyl dextran of CM7 biosensor chips according to the manufacturer's instructions. Experiments were performed at room temperature in PBS buffer with 0.005% (v/v) Tween‐20. Binding constants were obtained from a series of injections of pyruvate from 0.0625 × 10^–3^ to 8 × 10^–3^ m with a flow rate of 30 µL min^–1^. Binding affinity was estimated from the concentration dependence of the observed responses.

### Limited Proteolysis‐Small Molecule Mapping (LiP‐SMap)

Pyruvate solutions were prepared from ultrapure powders in HEPES buffer (25 × 10^–3^ m HEPES, 150 × 10^–3^ m NaCl, pH 7.4). The recombinant human GST‐SSRP1 proteins were expressed and purified from *Escherichia coli*. Purified GST‐SSRP1 proteins were aliquoted in equivalent volumes containing 100 µg of SSRP1 proteins and incubated for 20 min at 25 ℃ with or without pyruvate. Two final concentrations of pyruvate were set, 1 × 10^–3^ and 10 × 10^–3^ m, and three repetitions per group. Proteinase K (Sigma Aldrich) was added simultaneously to all the samples at a proteinase K to substrate mass ratio of 1:100 and incubated at 25 ℃ for 5 min. Digestion reactions were stopped by heating samples for 5 min at 98 ℃ in a thermocycler. Then the samples were reduced with 5 × 10^–3^ m TCEP (tris (2‐carboxyethyl) phosphine, Thermo) at 25 °C for 40 min followed by alkylation with 20 × 10^–3^ m IAA (iodoacetamide, Sigma Aldrich) at 25 °C for 30 min in the dark. Further digestion generating suitable peptides for shotgun proteome analysis was performed with Arg‐C (Promega) at 1:100 (Arg‐C: protein, w: w) overnight at 37 °C and then the reaction was stopped by adding formic acid to pH less than 2. Peptide mixtures were desalted with monospin C18 column (GL Sciences). Finally, the eluates were dried in a Speed Vacuum instrument and stored at ‐20 °C.

Samples were solubilized in 0.1% formic acid, and loaded onto a homemade 30 cm long pulled‐tip analytical column (ReproSil‐Pur C18 AQ 1.9 µm particle size, Dr. Maisch GmbH, 75 µm ID× 360 µm OD) connected to an Easy‐nLC1200 UHPLC (Thermo Scientific) for mass spectrometry analysis. The elution gradient and mobile phase constitution used for peptide separation were as follows: 0–15 min, 4%–8% B; 15–145 min, 8–30% B; 145–165 min, 30–60% B; 165–167 min, 60–100% B; 167–180 min, 100%–100% B (mobile phase A: 0.1% formic acid in water and mobile phase B: 0.1% formic acid in 80% acetonitrile) at a flow rate of 300 nL min^–1^. Peptides eluted from the LC column were directly electro‐sprayed into the mass spectrometer with the application of a distal 1.8 kV spray voltage. Survey full‐scan MS spectra (from m/z 300–1800) were acquired in the Orbitrap analyzer (Q Exactive) with resolution *r* = 70 000 at m/z 400. And top 20 MS/MS events were sequentially generated selected from the full MS spectrum at a 30% normalized collision energy. The dynamic exclusion time was 20 s.

The acquired MS/MS data were analyzed against a customized FASTA file which combined the human SSRP1 amino acid sequence downloaded from UniprotKB (www.uniprot.org) with a UniProtKB *Escherichia coli* database (released on Jan 04, 2019) to include all potential proteins in the samples by Andromeda algorithm built‐in MaxQuant engine (v1.6.0.13). Mass tolerances for precursor ions were set at 20 and 4.5 ppm for the first and main peptide search, respectively. Arg‐C was defined as a cleavage enzyme, the digestion mode was set as semispecific. Cysteine alkylation by iodoacetamide was specified as fixed modification with mass shift 57.02146; Methionine oxidation and protein N‐terminal acetylation were set as dynamic modification with mass shift 15.9949 and 42.0106. Label‐free quantification method was set as LFQ. A decoy database containing the reversed sequences of all the proteins was appended to the target database to accurately estimate peptide probabilities and false discovery rate (FDR), and FDR was set at 0.01. A built‐in label‐free quantification algorithm called LFQ was used for peptide quantification with a delayed normalization method^[^ ^47^ ^]^ to normalize peptide intensity quantification across all the samples. Quantified peptide data matrix was imported to limma package,^[^ ^48^ ^]^ implemented in the R/Bioconductor platform, for further data analysis. Linear fit model was used to fit the intensity of every peptide across all the samples for sake of good performance at filtering out significantly change peptides. Empirical Bayes moderated t‐tests were applied to calculate *p* value. The investigation aims at identifying conformotypic peptides that significantly change their abundance between the control and pyruvate treatment. Each condition with three biological replicates and statistically tested for differential conformotypic peptide abundances between conditions applying a logarithmic fold change cutoff of 1 and *p*‐value cutoff of 0.05. Peptide abundances statistics are obtained by grouping different precursor ions of the same peptide sequences.

### Histone Exchange Assays

Histone exchange assays were performed as described.^[^ ^27^ ^]^ Briefly, H2AX‐H2B heterodimers (Merck/Millipore) were first phosphorylated by purified recombinant ATM. Nucleosomes‐biotinylated (Active motif) were immobilized into streptavidin‐conjugated Dynabeads (Invitrogen) and then incubated with *γ*H2AX‐H2B, purified recombinant GST‐SPT16, GST‐SSRP1 WT or R54A in the presence or absence of pyruvate in reaction buffer (25 × 10^–3^ m HEPES [pH 7.6], 0.37 × 10^–3^ m EDTA, 0.35 × 10^–3^ m EGTA, 5 × 10^–3^ m MgCl_2_, 1 × 10^–3^ m DTT, 70 × 10^–3^ m KCl, 10% glycerol, 0.02% NP‐40, and 0.1 mg mL^–1^ BSA) for 1 h at 30 ℃. After washing three times with washing buffer (25 × 10^–3^ m HEPES [pH 7.6], 0.1 × 10^–3^ m EDTA, 0.5 × 10^–3^ m EGTA, 5 × 10^–3^ m MgCl_2_, 1 × 10^–3^ m DTT, 70 × 10^–3^ m KCl, 10% glycerol, 0.02% NP‐40, and 0.1 mg mL^–1^ BSA), the recruitment of *γ*H2AX to the chromatin was determined by IB analysis of immobilized nucleosomes with anti‐*γ*H2AX antibody.

### Intracranial Injection and Bioluminescence Imaging

Approximately 2 × 10^5^ (in 2 µL of DMEM per mouse) luciferase‐expressing PKM2‐depleted U87/EGFRvIII cells with reconstituted expression of rPKM2 WT/S222A or SSRP1‐depleted U87/EGFRvIII cells with reconstituted expression of rSSRP1 WT/R54A were intracranially injected into randomized 8 week old female athymic nude mice. Briefly, a small hand‐controlled twist drill that is 1 mm in diameter was used to make a hole in the animal's skull. The cell suspension was drawn up into the cuffed Hamilton syringe. The needle of the Hamilton syringe was slowly lowered into the central hole of the guide screw until the cuff rests on the screw surface. The cell suspension was slowly injected into the brain of mouse.^[^ ^49^ ^]^ And then the mice subjected to IR (X Ray, 6Gy) radiation in 4 and 8 d after inoculation. Five mice in each group were included. After inoculation, the mice were intraperitoneally injected with 100 µL of 15 mg mL^–1^ D‐luciferin (Xenogen) and subsequently anesthetized with isoflurane inhalation. Bioluminescence imaging with a CCD camera (IVIS Spectrum CT, PerkinElmer) was initiated 10 min after injection. All bioluminescent data were collected and analyzed using Living Image software (PerkinElmer). Survival durations of the implanted mice were compared. The use of mice was in compliance with ethical regulations and was approved by the institutional review board at Institute of Biochemistry and Cell Biology (PR01).

### Immunohistochemical Analysis

The tissue sections from paraffin‐embedded human GBM and astrocytoma specimens were stained with anti‐PKM2 pS222 antibody. The tissue sections were quantitatively scored according to the percentage of positive cells and staining intensity, as previously defined.^[^ ^39^ ^]^ The intensity of staining was rated on a scale of 0–3: 0, negative; 1, weak; 2, moderate; and 3, strong. The following proportion scores were assigned: 0 if 0% of the tumor cells showed positive staining, 1 if 0%–1% of cells were stained, 2 if 2%–10% were stained, 3 if 11%–30% were stained, 4 if 31%–70% were stained, and 5 if 71%–100% were stained. The proportion and intensity scores was then combined to obtain a total score (range, 0–8). Scores were compared with overall survival, defined as the time from the date of diagnosis to death or last known date of follow‐up. All patients received standard adjuvant radiotherapy after surgery, followed by treatment with an alkylating agent. The use of human brain tumor specimens and the database was approved by the Institutional Review Board at XinHua Hospital School of Medicine (XHEC‐F‐2021‐059). Informed consent was obtained from all patients.

### Statistical Analysis

No statistical methods were used to predetermine sample size. Mann Whitney test was used to analyze the statistical significance of the tail moments in comet assay between groups and *γ*H2AX or MDC1 foci number between groups (Figures 1B–D, 3C and 7B,C and Figures S1C,D, S3A,G and S7D,E, Supporting Information). A log‐rank test was used to analyze the statistical significance of the survival correlations between groups (Figures 5E, 7F and 8A). Other than the two analyses mentioned above, an unpaired, two‐tailed Student's t‐test was used for two‐group comparisons. *P* < 0.05 was considered to be significant. *P* < 0.01 was considered to be extremely significant.

## Conflict of Interest

The authors declare no conflict of interest.

## Author Contributions

S.W., R.C., and B.T. contributed equally to this work. W.Y. conceived this study. W.Y. and S.W. designed the study. S.W. performed most of the experiments. R.C. and S.W. performed the comet assay, immunofluorescence analysis and the animal experiments. P.W., C.P., and S.W. performed the LiP‐small molecule mapping. B.T. provided pathology assistance. H.G. provided the experimental assistance; J.L. provided conceptual advice; W.Y. and S.W. wrote the manuscript with comments from all authors.

## Supporting information

Supporting Information

## Data Availability

The data that support the findings of this study are available from the corresponding author upon reasonable request.
