# Supplementary material for: Pyruvate Facilitates FACT‐Mediated γH2AX Loading to Chromatin and Promotes the Radiation Resistance of Glioblastoma
Source: Adv Sci (Weinh). 2022 Jan 20;9(8):2104055. doi: 10.1002/advs.202104055 (PMC8922107; doi:10.1002/advs.202104055)
Supplement: Supplementary file 1 — Supporting Information [file ADVS-9-2104055-s001.pdf]

ADVANCED SCIENCE
Open Access

Supporting Information

for *Adv. Sci.*, DOI 10.1002/advs.202104055

Pyruvate Facilitates FACT-Mediated γH2AX Loading to Chromatin and Promotes the Radiation Resistance of Glioblastoma

Siyang Wu, Ruixiu Cao, Bangbao Tao, Ping Wu, Chao Peng, Hong Gao, Ji Liang and Weiwei Yang**

ADVANCED SCIENCE
Open Access

Supporting Information

for *Adv. Sci.,* DOI: 10.1002/advs.202104055

Pyruvate facilitates FACT-mediated γH2AX loading to chromatin and promotes the radiation resistance of glioblastoma

Siyang Wu, Ruixiu Cao, Bangbao Tao, Ping Wu, Chao Peng, Hong Gao, Ji Liang, Weiwei Yang**

Supplemental Information

Pyruvate facilitates FACT-mediated γH2AX loading to chromatin and promotes the radiation resistance of glioblastoma

Siyang Wu[1,2], Ruixiu Cao[1], Bangbao Tao[3], Ping Wu[4,5], Chao Peng[4,5], Hong Gao[1], Ji Liang[1*], Weiwei Yang[1,2*].

Figure S1

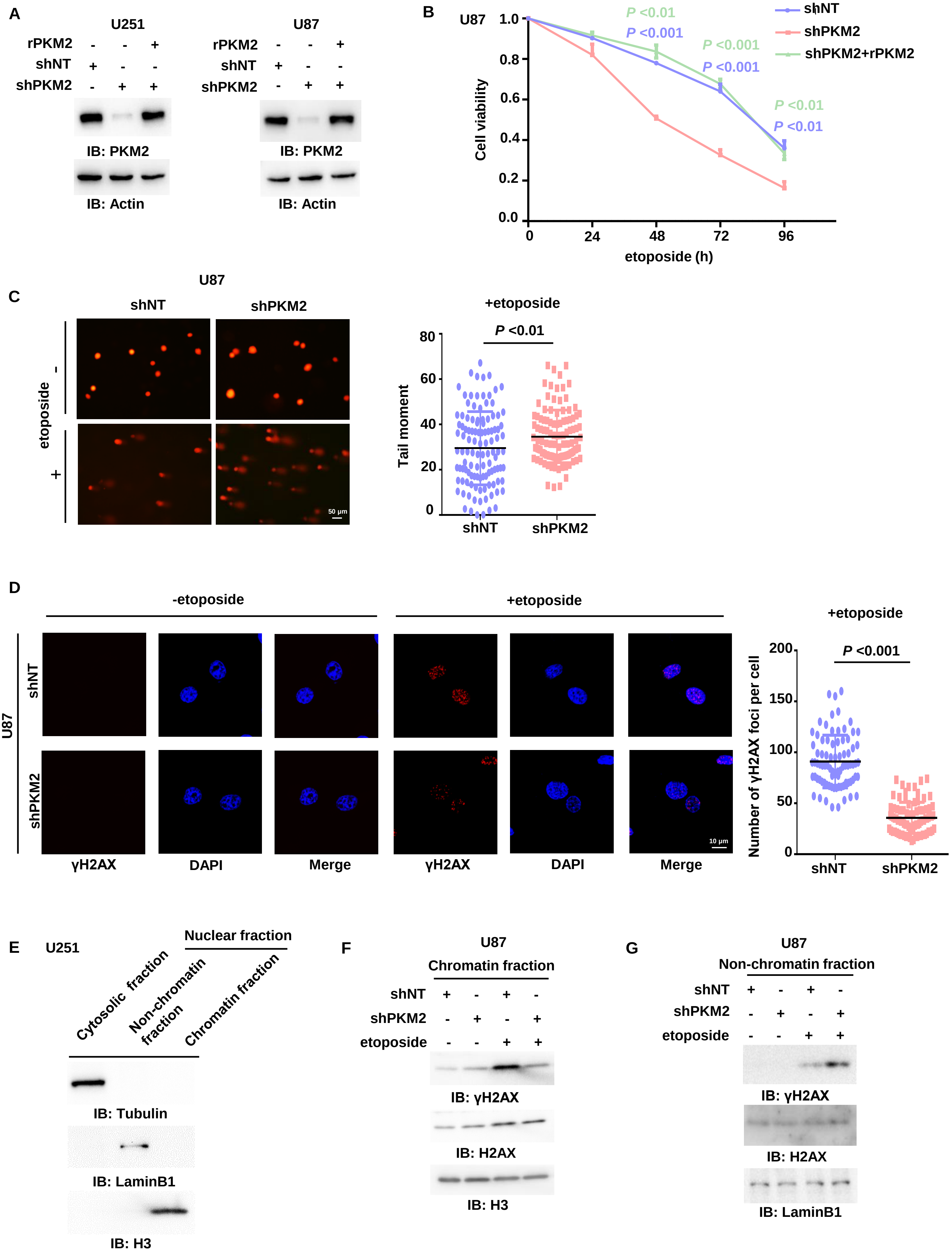
A
U251
U87
rPKM2
shNT
shPKM2
IB: PKM2
IB: Actin
B
U87
Cell viability
etoposide (h)
shNT
shPKM2
shPKM2+rPKM2
P <0.01
P <0.001
C
U87
shNT
shPKM2
etoposide
50 μm
+etoposide
P <0.01
Tail moment
D
-etoposide
+etoposide
U87
γH2AX
DAPI
Merge
10 μm
P <0.001
Number of γH2AX foci per cell
E
U251
Nuclear fraction
Cytosolic fraction
Non-chromatin fraction
Chromatin fraction
IB: Tubulin
IB: LaminB1
IB: H3
F
Chromatin fraction
etoposide
IB: γH2AX
IB: H2AX
G
Non-chromatin fraction

Figure S1. PKM2 is required for γH2AX levels in chromatin, DNA repair and tumor cell survival. Related to Figure 1.

IB analyses were performed with indicated antibodies. Data are representative of at least three independent experiments.

(A) U251 or U87 cells were depleted of endogenous PKM2 and rescued with or without shRNA-resistant (r) PKM2.

(B) U87 cells stably expressing shNT or shPKM2 were rescued with or without rPKM2. Cells were treated with etoposide (200 μM) for indicated time. Cell viability was determined using Trypan blue staining. Data represent the mean ± SD of the viability of the cells from three independent experiments (two-tailed Student's t-test). *P* values for comparisons between shNT and shPKM2 are shown in blue; comparisons between shPKM2 and shPKM2+rPKM2 are shown in green.

(C) U87 cells stably expressing shNT or shPKM2 were treated with or without etoposide (500 μM) for 3 h. The representative images of the comet assay in these cells were shown (left panel). Scatter dot plot (right panel) of the tail moments in the comet assay from shNT-expressing cells (n=112) or shPKM2-expressing cells (n=113) treated with etoposide. Data represent mean ± SD of the tail moments (Mann Whitney test). Data are representative of three independent experiments.

(D) U87 cells stably expressing shNT or shPKM2 were treated with or without etoposide (40 μM, 0.5 h). IF staining was performed using anti-γH2AX antibody. Representative images were shown (left panel). Scatter dot plot (right panel) of the number of γH2AX foci per cell. Data represent the mean ± SD of the number of γH2AX foci from shNT-expressing cells (n=81) and shPKM2-expressing cells (n=84) treated with etoposide (Mann Whitney test). Data are representative of three independent experiments.

(E) Cytosolic fraction, non-chromatin-bound fraction and chromatin fraction were prepared in U251 cells. Tubulin, LaminB1 or histone H3 was the marker for cytosolic fraction, non-chromatin nuclear fraction or chromatin fraction, respectively.

(F, G) U87 cells with or without PKM2 depletion were treated with or without etoposide (200 μM, 1 h). Chromatin fraction (F) and non-chromatin-bound fraction (G) were prepared.

Figure S2

A

Protein ID	Protein description	Gene name	Mol. weight [kDa]	Score	Intensity PKM2 Ctrl	Intensity PKM2 +Etoposide
Q9Y5B9	FACT complex subunit SPT16	SUPT16H	119.91	6.3855	0	2055500

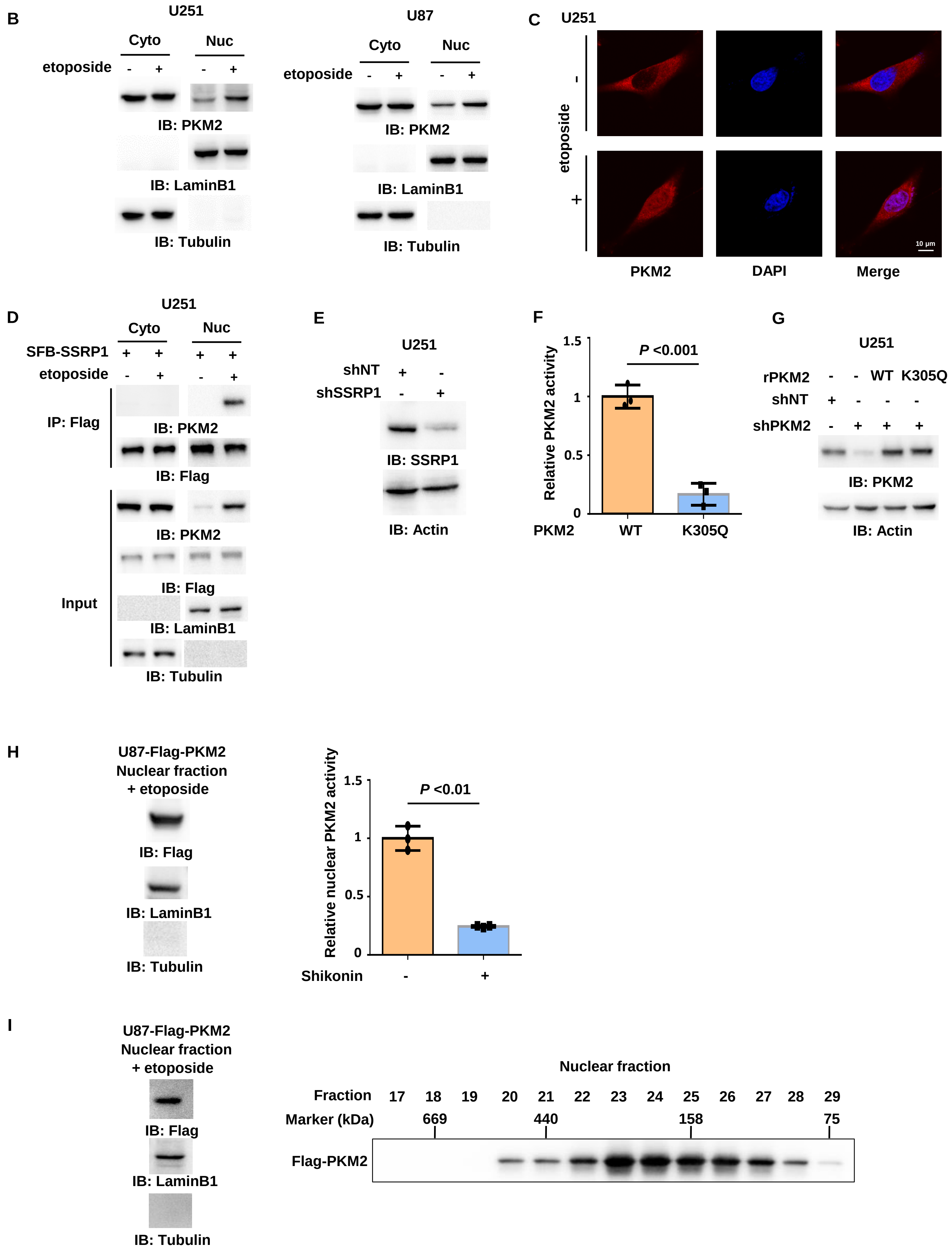

Figure S2. PKM2 interacts with FACT and enhances the interaction between FACT and non-chromatin bound γH2AX. Related to Figure 2.

IB analyses were performed with indicated antibodies. Data are representative of at least three independent experiments.

(A) Mass spectrometry analysis of PKM2-associated protein with or without etoposide treatment in U251 (200 μM, 1 h).

(B) U251 (left panel) or U87 (right panel) cells were treated with or without etoposide (200 μM, 1 h). Cytosolic (Cyto) and nuclear (Nuc) fractions were prepared.

(C) U251 cells were treated with or without etoposide (200 μM, 1 h). IF staining was performed using anti-PKM2 antibody.

(D) U251 cells stably expressing SFB-SSRP1 were treated with or without etoposide (200 μM, 1 h). Cytosolic (Cyto) and nuclear (Nuc) fractions were prepared and co-IP was performed with anti-Flag antibody

(E) U251 cells stably expressing shNT or shSSRP1 were generated.

(F) HEK293T cells were transfected with Flag-PKM2 WT or K305Q. Flag-PKM2 proteins were immunoprecipitated for PKM2 enzymatic activity assay. Data represent the mean ± SD of the relative PKM2 activity from three biologically independent experiments (two-tailed Student's t-test).

(G) PKM2-depleted U251 cells were rescued with rPKM2 WT or K305Q.

(H) U87 cells stably expressing Flag-PKM2 were treated with etoposide (200 μM, 1 h). Nuclear fractions were prepared in these cells (left panel). Flag-PKM2 was immunoprecipitated from the nuclear lysates. The glycolytic activity of nuclear PKM2 was determined in the absence or presence of Shikonin, the inhibitor of PKM2. Data represent the mean ± SD of the relative PKM2 activity from three biologically independent experiments (two-tailed Student's t-test).

(I) U87 cells stably expressing Flag-PKM2 were treated with etoposide (200 μM, 1 h). Nuclear fractions were prepared in these cells (left panel). Flag-PKM2 was immunoprecipitated from the nuclear fractions and eluted with flag peptides. The nuclear PKM2 were separated by gel filtration, followed by western analysis. Fraction numbers and elution of molecular weight markers (MW) are indicated.

Figure S3

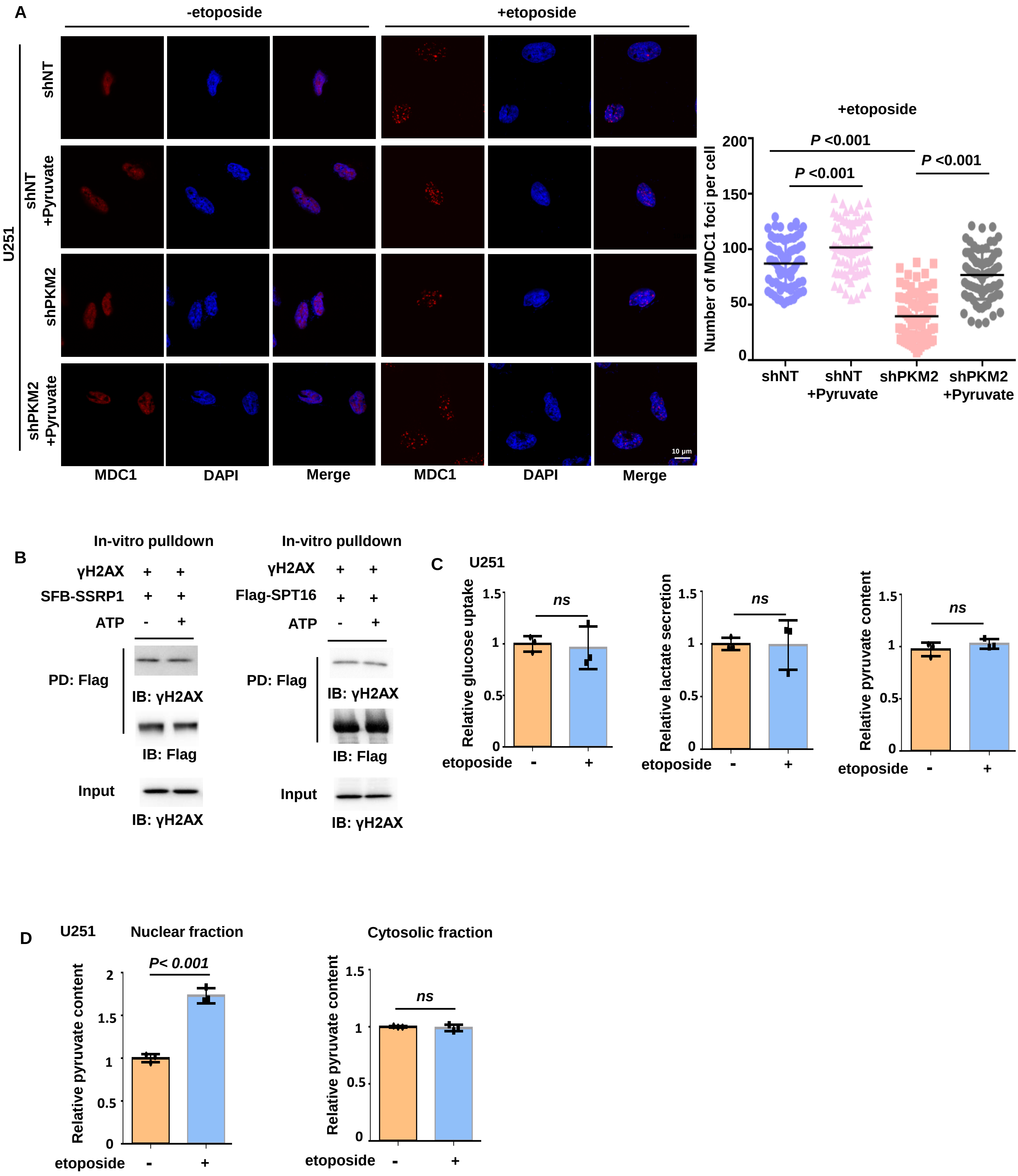

Next page ↓

Figure S3

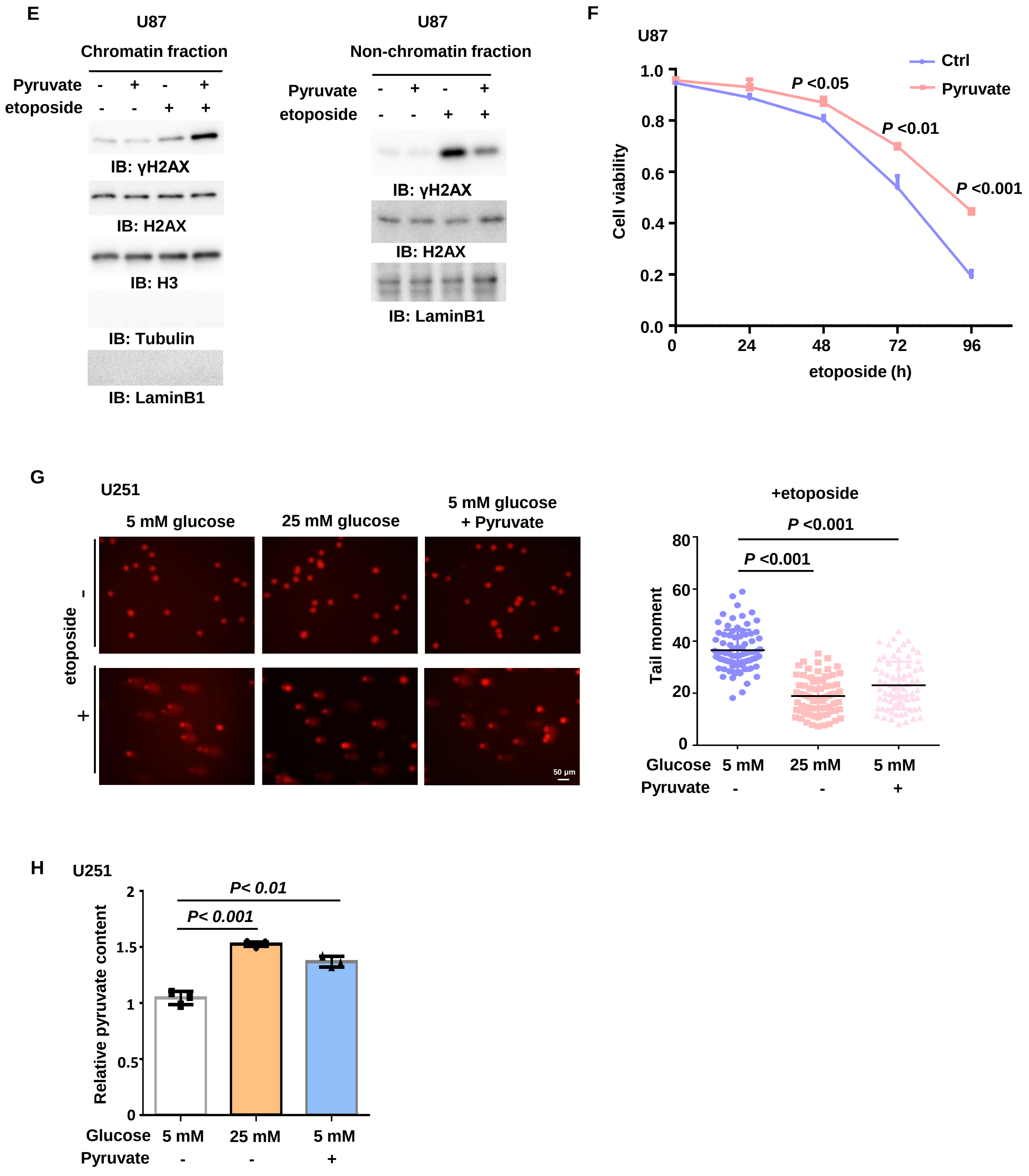
E
U87
Chromatin fraction
Pyruvate - + - +
etoposide - - + +
IB: γH2AX
IB: H2AX
IB: H3
IB: Tubulin
IB: LaminB1
U87
Non-chromatin fraction
Pyruvate - + - +
etoposide - - + +
IB: γH2AX
IB: H2AX
IB: LaminB1
F
U87
Ctrl
Pyruvate
Cell viability
P <0.05
P <0.01
P <0.001
1.0
0.8
0.6
0.4
0.2
0.0
0
24
48
72
96
etoposide (h)
G
U251
5 mM glucose
25 mM glucose
5 mM glucose
+ Pyruvate
etoposide
-
+
50 μm
+etoposide
P <0.001
P <0.001
Tail moment
80
60
40
20
0
Glucose 5 mM 25 mM 5 mM
Pyruvate - - +
H
U251
P< 0.01
P< 0.001
Relative pyruvate content
2
1.5
1
0.5
0
Glucose 5 mM 25 mM 5 mM
Pyruvate - - +

Figure S3. Pyruvate increases the interaction between FACT and γH2AX, γH2AX levels in chromatin and tumor cell survival upon DNA damage. Related to Figure 3.

IB analyses were performed with indicated antibodies. Data are representative of at least three independent experiments.

(A) U251 cells stably expressing shNT or shPKM2 were supplemented with or without pyruvate (10 mM, 3 h) and then treated with or without etoposide (40 μM, 0.5 h). IF staining was performed using anti-MDC1 antibody. Representative images were shown (left panel). Scatter dot plot (right panel) of the number of MDC1 foci per cell. Data represent mean ± SD of the MDC1 foci number of cells treated with etoposide, expressing shNT with (n=75) or without (n=78) pyruvate, or cells expressing shPKM2 with (n=76) or without (n=77) pyruvate (Mann Whitney test). Data are representative of three independent experiments.

(B) SSRP1 or SPT16 was immunoprecipitated and purified from U251 cells stably expressing SFB-SSRP1 or Flag-SPT16. γH2AX was immunoprecipitated and purified from etoposide-treated U251 cells. The in-vitro pulldown experiment was performed by incubating the purified SSRP1 or SPT16 with purified γH2AX in the absence or presence of ATP (1 mM).

(C) U251 cells were treated with etoposide (200 μM, 1 h). Glucose consumption, lactate secretion and pyruvate content were determined. Data represent the mean ± SD of three biologically independent experiments. (two-tailed Student's t-test).

(D) The levels of pyruvate in cytosol or nucleus from U251 cells after etoposide treatment (200 μM, 1h) were determined. Data represent the mean ± SD of the relative pyruvate content from three biologically independent experiments (two-tailed Student's t-test).

(E) U87 cells were supplemented with pyruvate (10 mM, 3 h) before etoposide (200 μM, 1 h) treatment. Chromatin (left panel) and non-chromatin-bound fractions (right panel) were prepared.

(F) U87 cells were supplemented with or without pyruvate (10 mM, 3 h) and then treated with etoposide (200 μM) for indicated time. Cell viability was determined. Data represent the mean ± SD of three biologically independent experiments. (two-tailed Student's t-test).

(G, H) U251 cells were cultured in low glucose (5 mM) DMEM media, high glucose (25mM) DMEM media or low glucose DMEM media supplemented with pyruvate (10 mM, 3h) for 24 h, the pyruvate content was detected (H). These cells were treated with or without etoposide (500 μM) for 3 h (G). The representative images of the comet assay in these cells were shown (left panel). Scatter dot plot (right panel) represents the tail moments in the comet assay from cells cultured with low glucose DMEM (n=82), cells cultured with high

glucose DMEM (n=75) and cells cultured with low glucose DMEM supplemented with pyruvate (n=76) treated with etoposide. Data represent mean ± SD of the tail moments (Mann Whitney test). Data are representative of three independent experiments.

Figure S4

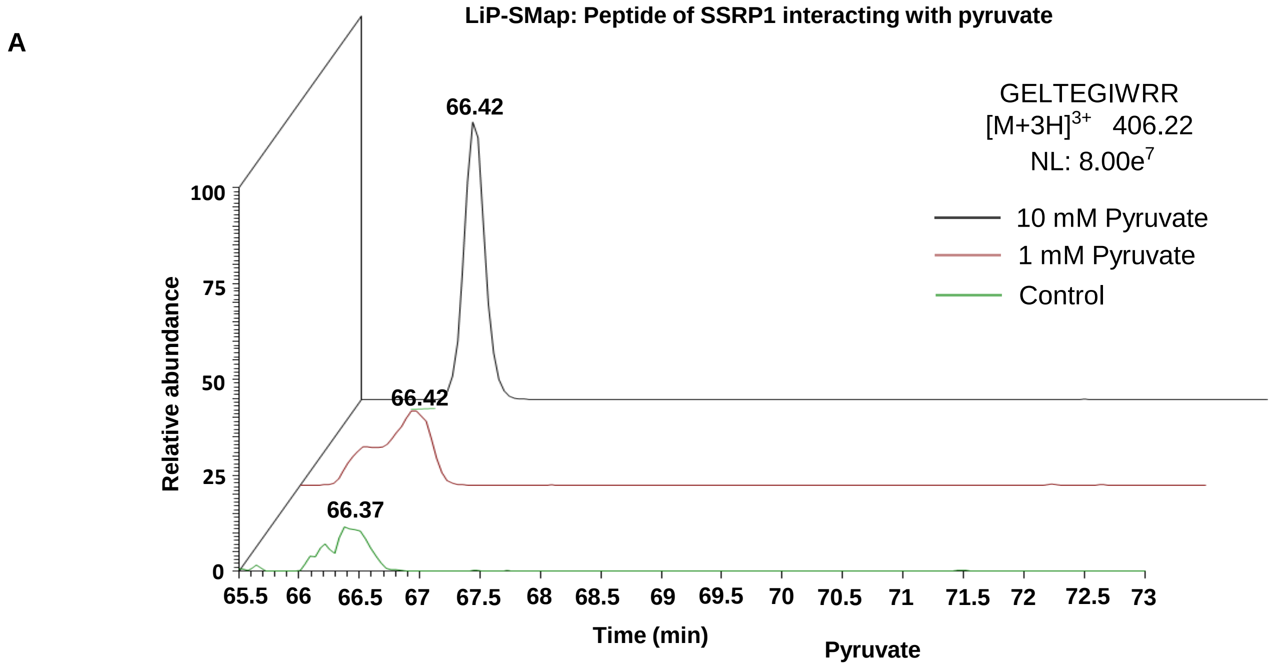

Sequence	Location	Pyruvate 1 mM logFC	Pyruvate 1 mM *P*.Value	Pyruvate 10 mM logFC	Pyruvate 10 mM *P*.Value
GELTEGIWRR	46-55	0.640	0.143	1.160	0.010

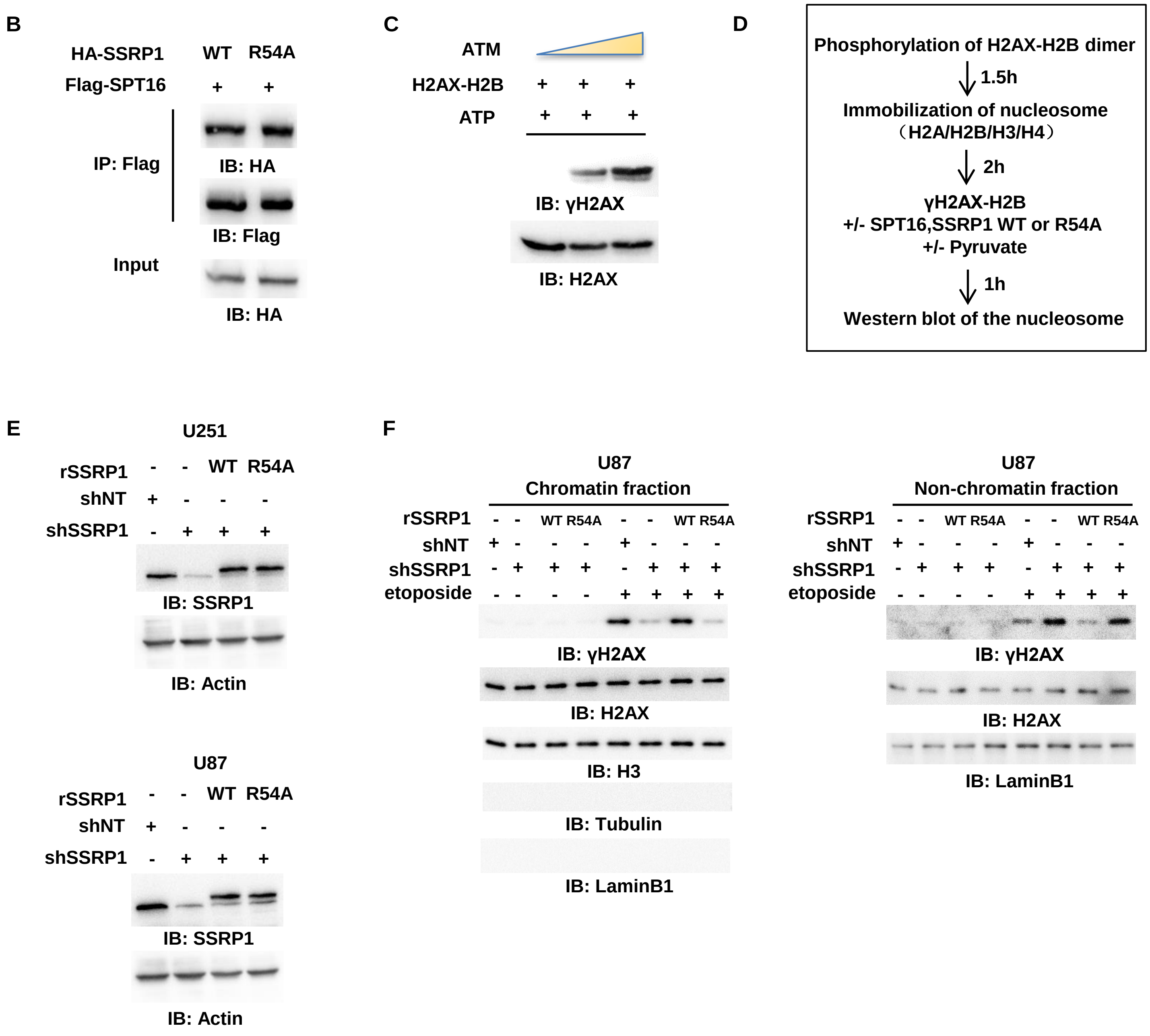

Figure S4. The binding of pyruvate to SSRP1 is required for FACT-mediated γH2AX loading to chromatin. Related to Figure 4.

IP and IB analyses were performed with indicated antibodies. Data are representative of at least three independent experiments.

(A) Pyruvate-associated peptide in SSRP1 identified by Limited proteolysis-small molecule mapping (LiP-SMap). logFC: a logarithmic fold change of pyruvate vs control. NL: normalized intensity which stands for the 100 percent of the intensity is 8.00e7.

(B) HEK293T cells were transfected with Flag-SPT16 and HA-SSRP1 WT or R54A.

(C) In vitro kinase assay was carried out with purified recombinant monomeric ATM and commercially purchased recombinant H2AX-H2B dimer.

(D) Schematic diagram of the in vitro histone exchange assay.

(E) SSRP1-depleted U251 (top panel) and U87 (bottom panel) cells were rescued with rSSRP1 WT or R54A.

(F) U87 cells were infected with the lentivirus expressing shNT or shSSRP1 and the SSRP1-depleted U87 cells were reconstituted with the expression of rSSRP1 WT or R54A. These cells were then treated with or without etoposide (200 μM, 1 h). Chromatin fraction (left panel) and non-chromatin-bound fraction (right panel) were prepared.

Figure S5

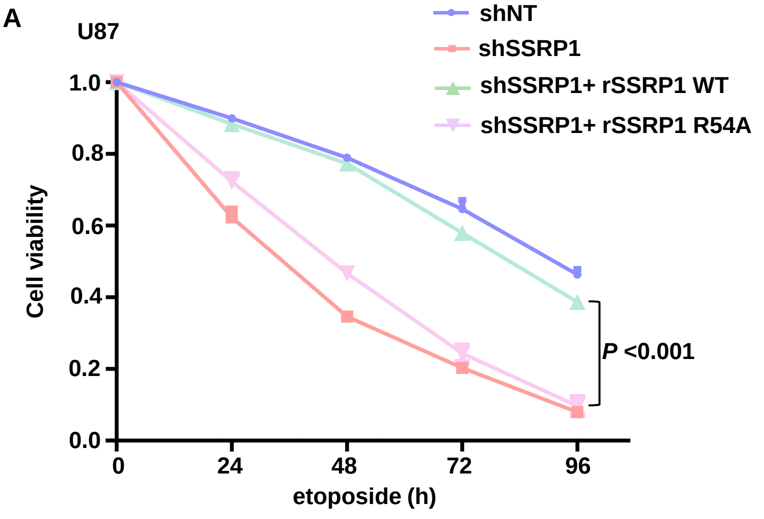
A
U87
shNT
shSSRP1
shSSRP1+ rSSRP1 WT
shSSRP1+ rSSRP1 R54A
Cell viability
1.0
0.8
0.6
0.4
0.2
0.0
0
24
48
72
96
etoposide (h)
P <0.001

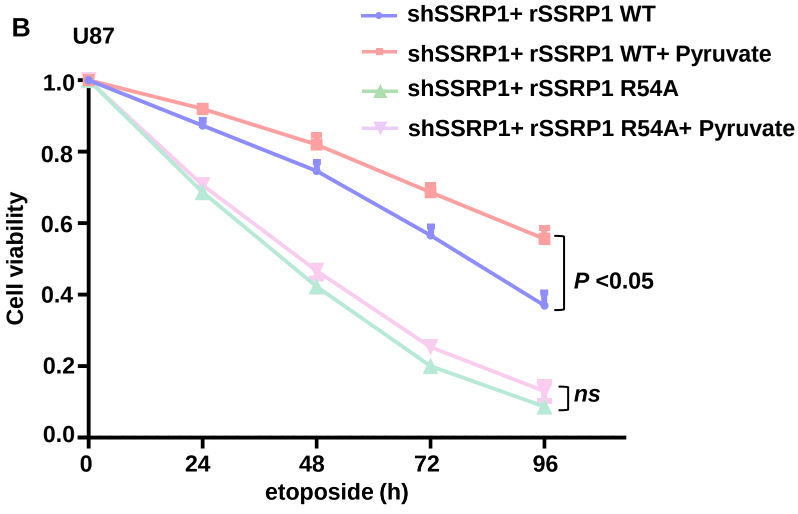
B
U87
shSSRP1+ rSSRP1 WT
shSSRP1+ rSSRP1 WT+ Pyruvate
shSSRP1+ rSSRP1 R54A
shSSRP1+ rSSRP1 R54A+ Pyruvate
Cell viability
1.0
0.8
0.6
0.4
0.2
0.0
0
24
48
72
96
etoposide (h)
P <0.05
ns

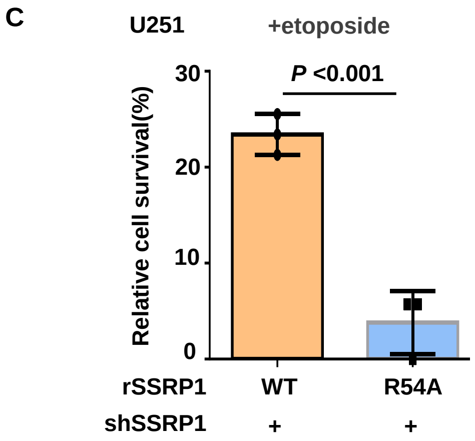
C
U251
+etoposide
P <0.001
Relative cell survival(%)
30
20
10
0
rSSRP1
WT
R54A
shSSRP1
+
+

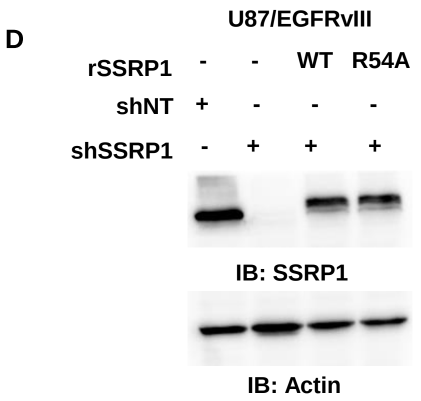
D
U87/EGFRvIII
rSSRP1 - - WT R54A
shNT + - - -
shSSRP1 - + + +
IB: SSRP1
IB: Actin

Figure S5. The binding of pyruvate to SSRP1 is required for tumor cell survival and irradiation resistance of glioblastoma. Related to Figure 5.

IB analyses were performed with indicated antibodies. Data are representative of at least three independent experiments.

(A) SSRP1-depleted U87 cells reconstituted with rSSRP1 WT or R54A were treated with etoposide (200 μM) for indicated time. Cell viability was determined. Data represent the mean ± SD of the viability of the cells from three independent experiments (two-tailed Student's t-test). *P* values for comparisons between shSSRP1+rSSRP1 WT and shSSRP1+rSSRP1 R54A are shown.

(B) SSRP1-depleted U87 cells reconstituted with rSSRP1 WT or R54A were supplemented with or without pyruvate (10 mM, 3 h) and then treated with etoposide (200 μM) for indicated time. Cell viability was determined. Data represent the mean ± SD of the viability of the cells from three independent experiments (two-tailed Student's t-test).

(C) SSRP1-depleted U251 cells reconstituted with rSSRP1 WT or R54A were split by the limited dilution and treated with etoposide (50 μM). 9 days later, the dishes were stained with crystal violet and visible colonies were counted. Data represent the mean ± SD of the viability of the cells from three independent experiments (two-tailed Student's t-test).

(D) SSRP1-depleted U87/EGFRvIII cells were rescued with rSSRP1 WT or R54A.

Figure S6

A

PTM Sites	Localization score	Modified spec count/ unmodified spec count
S222	1000	1/133
Y390	342.54	3/356
T405	9.64	1/259
Y175	7.76	1/134

C

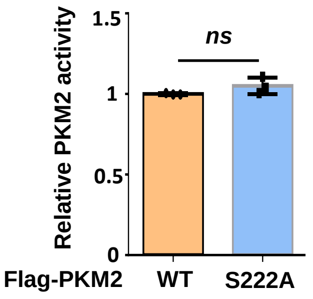

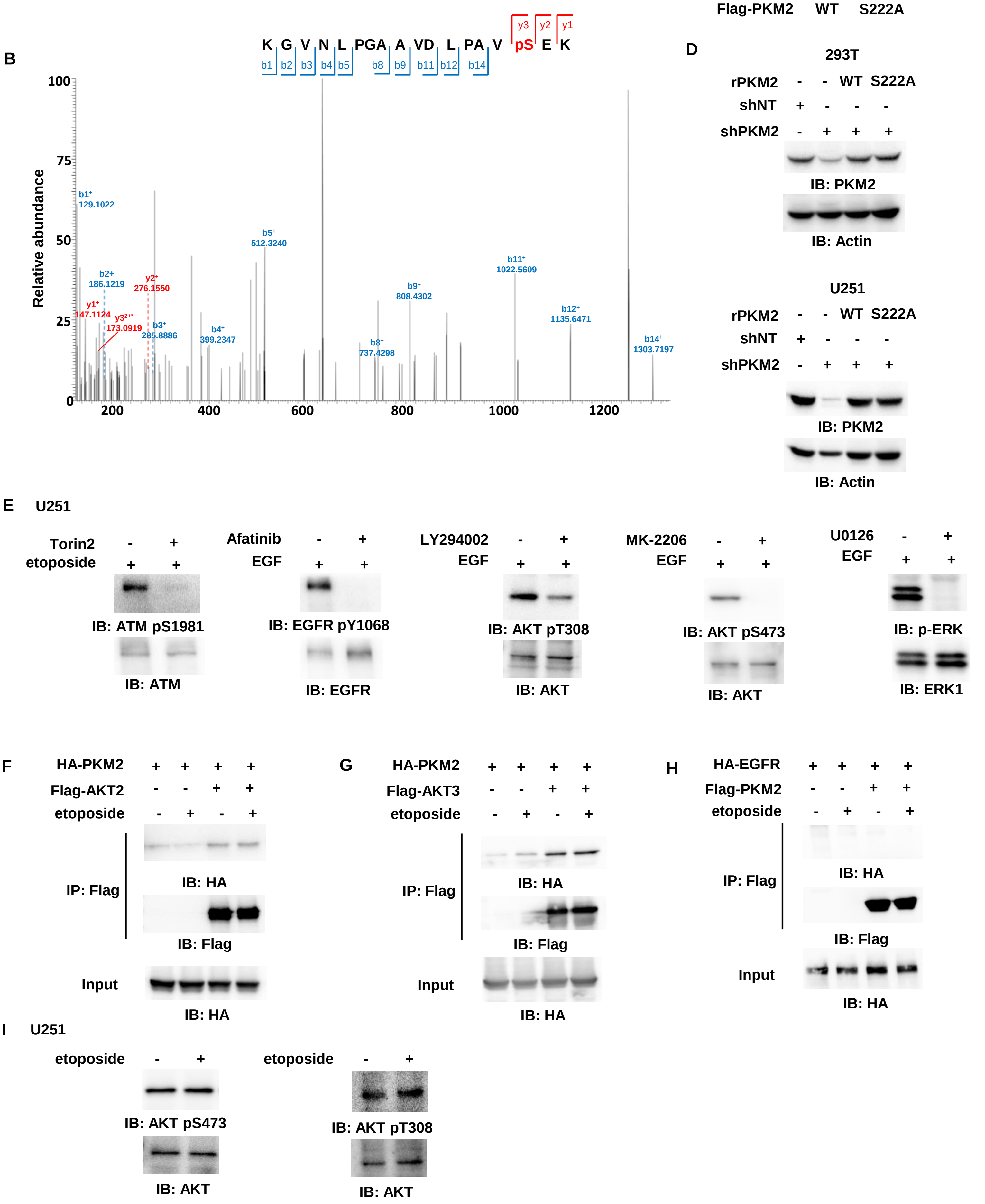

Figure S6. AKT1-dependent PKM2 S222 phosphorylation is necessary for PKM2 and FACT interaction upon DNA damage. Related to Figure 6.

IB and IP analyses were performed with indicated antibodies. Data are representative of at least three independent experiments.

(A) Mass spectrometry analyses of PKM2 phosphorylation sites after etoposide treatment (200 μM, 1 h). Localization score: a phosphorylation site confidence index. A value greater than 13 indicates a phosphorylation possibility of 95%, and a value greater than 19 indicates a phosphorylation possibility of 99%. The spec count can be used to estimate the relative content of the peptide.

(B) The figure shows a higher-energy collisional dissociation (HCD) MS/MS spectrum recorded on the $[M+2H]^{2+}$ ion at m/z 922.98 of the human PKM2 peptide KGVNLPGAAVDLPAVSEK harboring one phosphorylated site (denoted by pS). Predicted b- and y-type ions (not including all) are listed above and below the peptide sequence, respectively, the asterisk symbol means the neutral loss of phosphate group. Ions observed are labeled in the spectrum and indicate that S222 of the protein is phosphorylated.

(C) The enzymatic activity of Flag-PKM2 proteins precipitated from U251 cells expressing Flag-PKM2 WT or S222A was examined. Data represent the means ± SD of relative PKM2 activity from three independent experiments (two-tailed Student's t-test).

(D) PKM2-depleted HEK293T and U251 cells were rescued with rPKM2 WT or S222A.

(E) U251 cells were treated with or without ATM/DNA-PK inhibitor Torin2 (100 nM), EGFR inhibitor Afatinib (5 μM), PI3K inhibitor LY294002 (1 μM), AKT inhibitor MK-2206 (1 μM) or MEK inhibitor U0126 (5 μM) for 12 h.

(F) Co-IP was performed with anti-Flag antibody in HEK293T cells transfected with HA-PKM2 and Flag-AKT2 after etoposide treatment (200 μM, 1 h).

(G) Co-IP was performed with anti-Flag antibody in HEK293T cells transfected with HA-PKM2 and Flag-AKT3 after etoposide treatment (200 μM, 1 h).

(H) Co-IP was performed with anti-Flag antibody in HEK293T cells transfected with HA-EGFR and Flag-PKM2 after etoposide treatment (200 μM, 1 h).

(I) U251 cells were treated with or without etoposide (200 μM, 1 h).

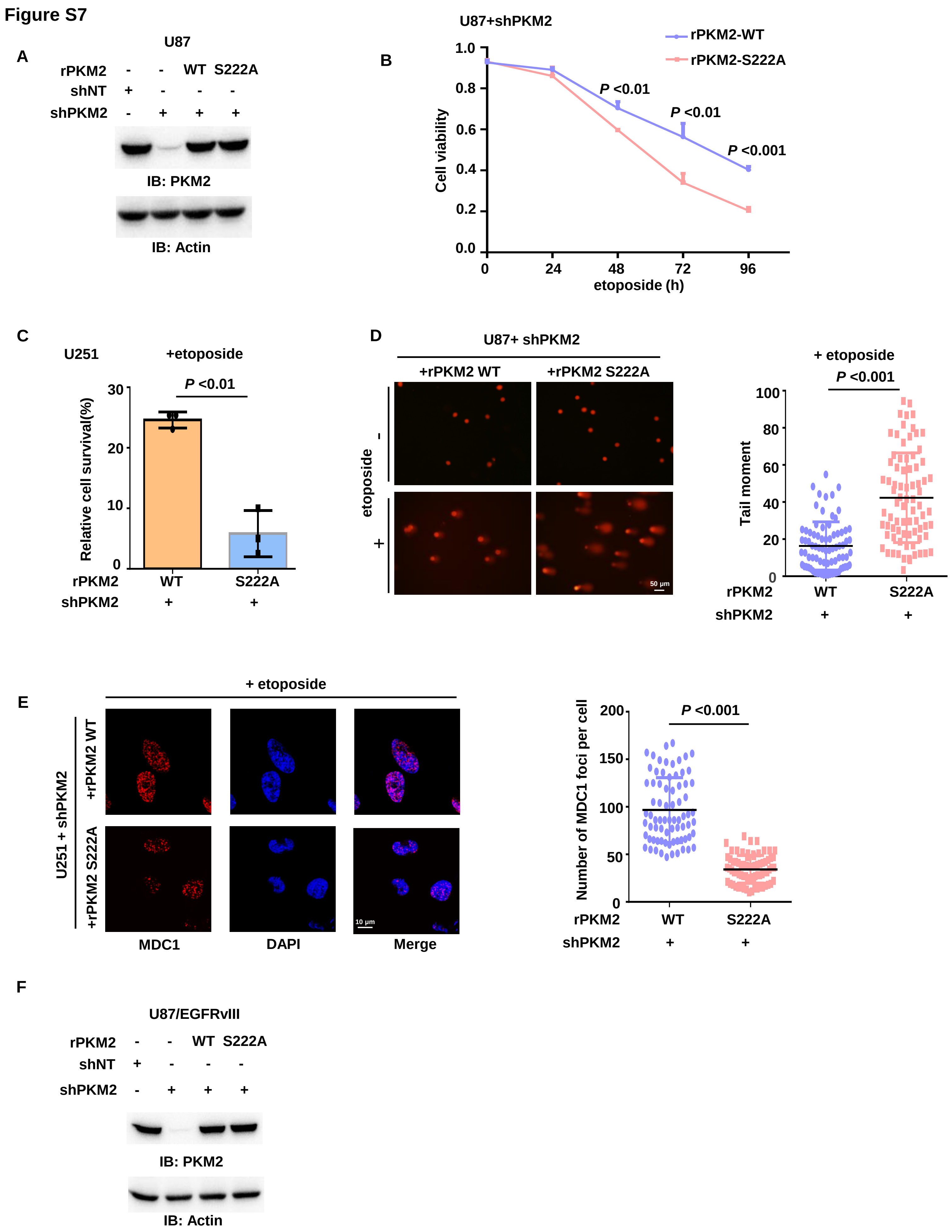
Figure S7
A
U87
rPKM2 - - WT S222A
shNT + - - -
shPKM2 - + + +
IB: PKM2
IB: Actin
B
U87+shPKM2
rPKM2-WT
rPKM2-S222A
Cell viability
1.0
0.8
0.6
0.4
0.2
0.0
P <0.01
P <0.01
P <0.001
0 24 48 72 96
etoposide (h)
C
U251
+etoposide
P <0.01
Relative cell survival(%)
30
20
10
0
rPKM2 WT S222A
shPKM2 + +
D
U87+ shPKM2
+rPKM2 WT
+rPKM2 S222A
etoposide
-
+
50 μm
+ etoposide
P <0.001
Tail moment
100
80
60
40
20
0
rPKM2 WT S222A
shPKM2 + +
E
+ etoposide
U251 + shPKM2
+rPKM2 WT
+rPKM2 S222A
10 μm
MDC1
DAPI
Merge
Number of MDC1 foci per cell
200
150
100
50
0
P <0.001
rPKM2 WT S222A
shPKM2 + +
F
U87/EGFRvIII
rPKM2 - - WT S222A
shNT + - - -
shPKM2 - + + +
IB: PKM2
IB: Actin

Figure S7. PKM2 pS222 is required for DNA repair, tumor cell survival and radiation resistance of glioblastoma. Related to Figure 7.

IB analyses were performed with indicated antibodies. Data are representative of at least three independent experiments.

(A-B) (A) PKM2-depleted U87 cells were rescued with rPKM2 WT or S222A. (B) These cells were treated with etoposide (200 μM) for the indicated time. Cell viability was determined. Data represent the means ± SD of the viability of the cells from three independent experiments (two-tailed Student's t-test).

(C) PKM2-depleted U251 cells reconstituted with rPKM2 WT or R54A were split by the limited dilution and treated with etoposide (50 μM). 9 days later, the dishes were stained with crystal violet and visible colonies were counted. Data represent the mean ± SD of the viability of the cells from three independent experiments (two-tailed Student's t-test).

(D) PKM2-depleted U87 cells were rescued with rPKM2 WT or S222A and then treated with or without etoposide (500 μM, 3 h). The representative images of the comet assay in these cells were shown (left panel). Scatter dot plot (right panel) of the tail moments in the comet assay from shPKM2+rPKM2 WT cells (n=79) or shPKM2+rPKM2 S222A cells (n=79) treated with etoposide. Data represent mean ± SD of the tail moments (Mann Whitney test). Data are representative of three independent experiments.

(E) PKM2-depleted U251 cells were rescued with rPKM2 WT or S222A. Cells were treated with etoposide (40 μM, 0.5 h). IF staining was performed using anti-MDC1 antibody. Representative images were shown (left panel). Scatter dot plot (right panel) of the number of MDC1 foci per cell. Data represent the mean ± SD of the number of MDC1 foci from shPKM2+rPKM2 WT cells (n=75) and shPKM2+rPKM2 S222A cells (n=74) treated with etoposide (Mann Whitney test). Data are representative of three independent experiments.

(F) PKM2 was depleted in U87/EGFRvIII cells stably expressing luciferase, followed by reconstituted expression of rPKM2 WT or S222A.

Figure S8

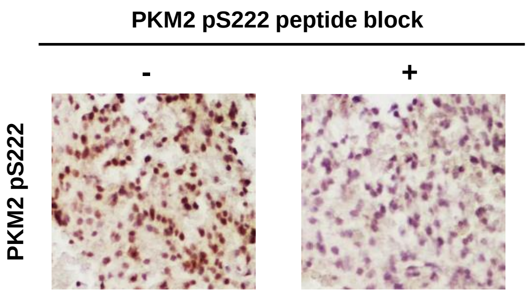

Figure S8. Validation of the PKM2 pS222 antibody specificity. Related to Figure 8.

IHC analyses of human GBM tissues were performed with the anti-PKM2 pS222 antibody in the absence or presence of specific blocking peptides.
